# CXCR4 over-expression and survival in cancer: A system review and meta-analysis

**DOI:** 10.18632/oncotarget.3217

**Published:** 2014-12-31

**Authors:** Hongli Zhao, Liyuan Guo, Hong Zhao, Jiaxin Zhao, Hao Weng, Bin Zhao

**Affiliations:** ^1^ Department of Medical Oncology, The Third Affiliated Hospital of Harbin Medical University, Harbin, China; ^2^ Department of Gynecological Oncology, The Third Affiliated Hospital of Harbin Medical University, Harbin, China; ^3^ Department of Neurosurgery, The Fourth Affiliated Hospital of Harbin Medical University, Harbin, China; ^4^ Shanghai Jiaotong University Affiliated Sixth People's Hospital South Campus, Shanghai, China; ^5^ Harbin Medical University, Daqing Campus, China

**Keywords:** CXCR4, cancer, survival, prognostic biomarker, meta-analysis

## Abstract

C-X-C chemokine receptor 4 (CXCR4) is frequently over-expressed in various types of cancer; many agents against CXCR4 are in clinical development currently despite variable data for the prognostic impact of CXCR4 expression. Here eighty-five studies with a total of 11,032 subjects were included to explore the association between CXCR4 and progression-free survival (PFS) or overall survival (OS) in subjects with cancer. Pooled analysis shows that CXCR4 over-expression is significantly associated with poorer PFS (HR 2.04; 95% CI, 1.72-2.42) and OS (HR=1.94; 95% CI, 1.71-2.20) irrespective of cancer types. Subgroup analysis indicates significant association between CXCR4 and shorter PFS in hematological malignancy, breast cancer, colorectal cancer, esophageal cancer, renal cancer, gynecologic cancer, pancreatic cancer and liver cancer; the prognostic effects remained consistent across age, risk of bias, levels of adjustment, median follow-up period, geographical area, detection methods, publication year and size of studies. CXCR4 over-expression predicts unfavorable OS in hematological malignancy, breast cancer, colorectal cancer, esophageal cancer, head and neck cancer, renal cancer, lung cancer, gynecologic cancer, liver cancer, prostate cancer and gallbladder cancer; these effects were independence of age, levels of adjustment, publication year, detection methods and follow-up period. In conclusion, CXCR4 over-expression is associated with poor prognosis in cancer.

## INTRODUCTION

Cancer is a major public health problem globally. It is estimated that 12.7 million cancer cases and 7.6 million cancer deaths occurred in 2008 [1], and these numbers will continue to increase because of the aging and growth of the world population along with the overwhelming adoption of cancer-causing behaviors, like smoking, the consumption of high-fat diets and physical inactivity, in economically developing countries. Combination of surgery, radiotherapy and chemotherapy remains the standard treatment in most cancer cases; however, not all patients derive beneﬁt from it. Therefore, it is of great clinical value to identify applicable prognostic biomarkers, not only improving poor prognosis but also providing novel therapeutic targets. In the past decade, growing appreciation of the role of microenvironment in driving cancer cell biology has improved the understanding of oncologic disease. C-X-C chemokine receptor 4 (CXCR4) is believed to be one key factor in the cross-talking between cancer cells and its microenvironment, what makes it a very promising prognostic biomarker and target for cancer therapy [2].

CXCR4, a G-protein coupled chemokine receptor encoded on chromosome 2 [3], exerts its biological effect by binding stromal cell-derived factor 1 (SDF-1) [4]; recent evidence demonstrates ubiquitin, a small (76-amino acid) protein highly conserved among eukaryotic cells, is also a natural ligand of CXCR4 [5]. The expression of CXCR4 is low or absent in many healthy tissues, but it is demonstrated that CXCR4 is highly expressed in various different tumor types and has been considered the most widely expressed chemokine receptor in cancer [6]. Additionally, over-expression of CXCR4 in cancer specimens is associated with chemotaxis, invasion, angiogenesis and proliferation independent of their specific histological findings [7]. The important roles of CXCR4 in multiple diseases have encouraged the development of clinically viable CXCR4 antagonists, and resulted in the US Food and Drug Administration (FDA) approval of the first CXCR4 antagonist, plerixafor for patients with non-Hodgkin's lymphoma and multiple myeloma [2].

Despite the clinical development of anti-CXCR4 therapies, the prognostic value of CXCR4 over-expression across different tumors still remains unclear. Many studies have provided an insignificant association between CXCR4 expression and clinical outcome [8-11]. An improved understanding of this issue could have important public health and clinical implication considering many tumors are still incurable. With recently accumulating evidence, our goal here, therefore, was to evaluate the association between CXCR4 over-expression and progression-free survival (PFS) and overall survival (OS) by conducting a meta-analysis among patients with different types of cancer, thereby allowing more rational development of therapeutic strategies against this receptor.

## RESULTS

### Eligible studies

**Figure 1:**
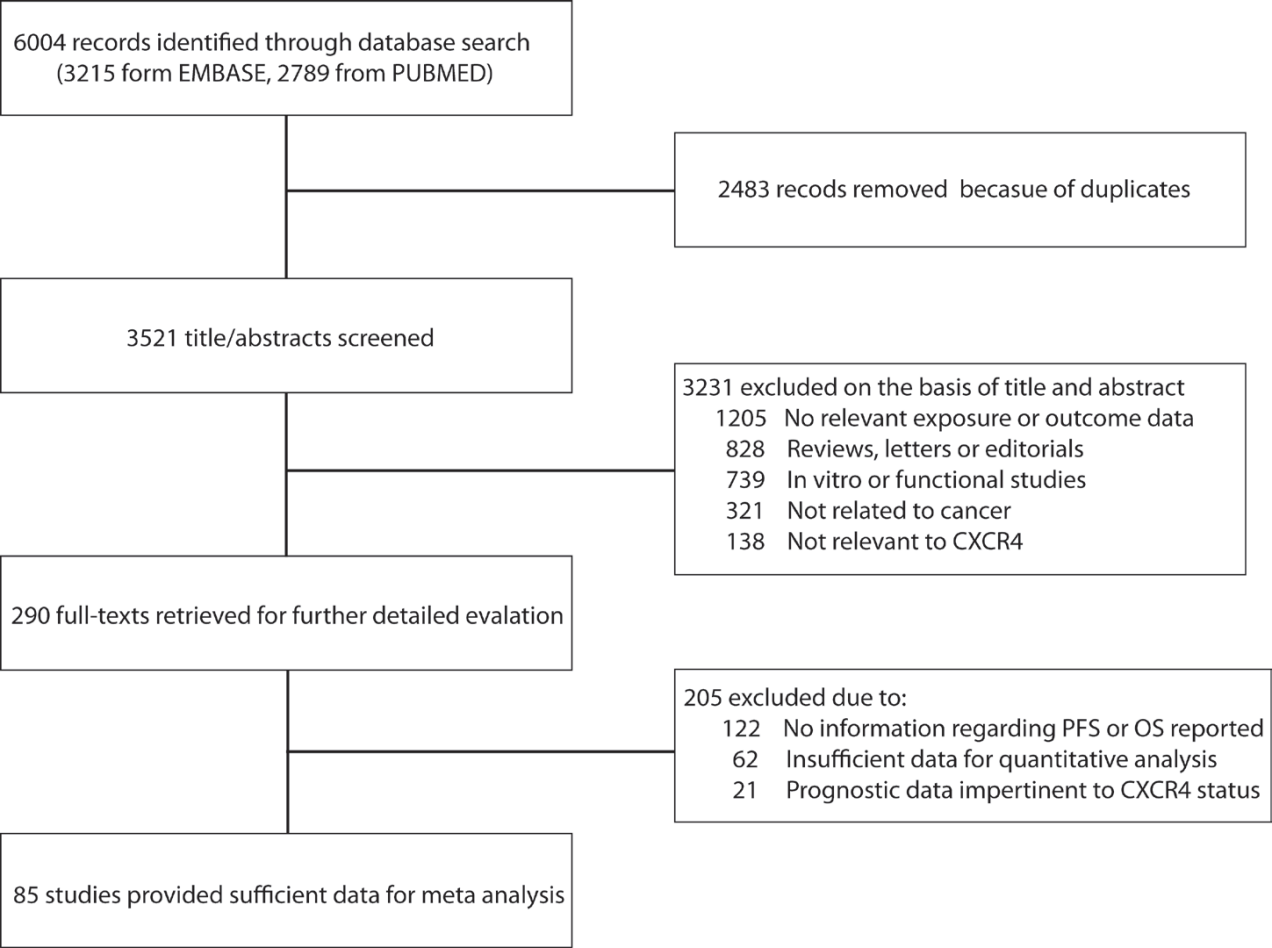
Using standardized protocol for a comprehensive search, a total of 85 studies were included for current meta-analysis

The search strategy identified 3521 unique citations. After initial screening based on titles and abstracts, 3231 studies were excluded. The main reasons for exclusion were failure to examine disease prognosis or the studies were not original studies. Of the 290 articles remained for further evaluation, 205 were excluded in the subsequent detailed assessments for no related information regarding PFS and OS or insufficient data for quantitative analysis. The remaining 85 reports met our inclusion criteria and were included in this meta-analysis. A ﬂow chart showing the study selection was presented in Figure 1.

### Study characteristics

The characteristics of the selected 85 studies are presented in Supplement table 1-3. These papers were published between 2004 and 2014 and all studies used retrospective cohort designs. A total of 11,032 participants were analyzed for CXCR4 status and its relationship to disease prognosis, of which 5971 (54%) were classified as CXCR4 over-expression. The average number of patients for these studies was 130, the average age was 58 years old and the mean follow-up was 53 months (Supplement table 1). The overall quality of the included studies was examined, 46 (54%) studies were at low risk of bias, and the rest 39 (46%) were at high risk of bias (Supplement table 2). Of all the 85 studies, 31 (36%) had their outcomes adjust for covariates (Supplement table 3). Geographically, 18 (22%) studies were conducted in the US and Canada, 25 (29%) in Europe, 41 (48%) in Asia, and 1(1%) in South America. In these 85 studies, 65 (76%) investigations detected the CXCR4 status by immunohistochemistry (IHC), 8 (9%) studies used polymerase chain reaction (PCR), 7 (8%) papers used western blot (WB), and the remaining 5 (6%) researches detected CXCR4 by fluorescence-activated cell sorting (FACS). Among 65 studies using IHC, the cutoff values were determined by percentage of CXCR4-positive cells in 23 studies (27%), by staining intensity scores in 20 studies (24%), and the cutoff points of rest 22 papers (26%) were based on both staining intensity score and percentage of CXCR4 positive cells.

Hematological malignancy (7 studies, 764 patients) [12-18], breast cancer (18 studies, 4125 patients) [10, 11, 19-34], colorectal cancer (7 studies, 515 patients) [35-41], esophageal cancer (7 studies, 886 patients) [42-48], gastric cancer (5 studies, 755 patients) [49-53], head and neck cancer (7 studies, 577 patients) [54-60], renal cancer (6 studies, 764 patients) [61-66], lung cancer (7 studies, 727 patients) [8, 67-72], melanoma (4 studies, 168 patients) [73-76], gynecologic cancer (8 studies, 826 patients) [9, 77-83], pancreatic cancer (2 studies, 320 patients) [84, 85], prostate cancer (2 studies, 109 patients) [86, 87], liver cancer (2 studies, 256 patients) [88, 89], sarcoma (2 studies, 168 patients) [90, 91] and gallbladder cancer (1 study, 72 patients) [92] were evaluated in current meta-analysis as shown in Table 1.

**Table 1:** Summary of 15 types of cancer studies included in current meta-analysis IHC, immunohistochemistry; WB, western blot; RCR, polymerase chain reaction; FACS, fluorescence-activated cell sorting.

Disease	No.of studies	Subtype	Location	Detetction method	No.of patients (CXCR4: +/−)	Average size of studies(range)	Average size of CXCR4+ patients(range)	Average age(years)	Average follow-up(months)
Hematological malignancy	7	Acute myeloid leukemia,5 (n=456); Myelodysplastic syndrome,1 (n=81); multiple myeloma,1 (n=227)	North america, 4 (n=366); Europe, 1 (n=90); Asia, 2（n=308）	IHC, 3 (n=276); FACS, 4 (n=488)	764(413/351)	109(53-227)	59(26-98)	57	13
Breast cancer	18	Triple negative breast cancer,3 (n=374); Node-positive breast cancer,2 (n=318); Node-negative breast cancer,2 (n=295); Locally advanced breast cancer, 2 (n=131); Inflammatory breast cancer, 1 (n=44); HER-2 negative breast cancer, 2 (n=218); Bilateral breast cancer, 1 (n=33); General breast cancer,5 （n=2712）	North america, 9 (n=1066); Europe, 5 (n=2603); Asia, 4（n=456）	IHC, 11 (n=3339); WB, 7 (n=786)	4125(1933/2192)	229(34-1382)	108(13-969)	53	63
Colorectal cancer	7	Colon cancer, 1 (n=125), Rectal cancer, 2 (n=121), Colorectal cancer, 4 (n=142)	North america, 1 (n=35); Europe, 3 (n=210); Asia, 3（n=270）	IHC, 4 (n=357); PCR, 3 (n=158)	515(288/227)	74(35-125)	41(16-74)	63	63
Esophageal Cancer	7	None	Europe, 2 (n=238); Asia, 5（n=648）	IHC, 7 (n=886)	886(566/320)	127(24-207)	81(13-174)	62	42
Gastric cancer	5	None	South america, 1 (n=104); Asia,4（n=651）	IHC, 5 (n=755)	755(361/394)	151(26-307)	72(13-112)	61	71
Head and neck cancer	7	Nasopharyngeal cancer, 2 (n=270); Oral cancer, 2 (n=133), Tongue cancer, 1(n=47), General head and neck cancer, 2 (n=127) Clear cell renal cancer,2 (n=329);	Europe, 2 (n=118); Asia,5（n=459）	IHC, 6 (n=506); PCR, 1 (n=71)	577(283/294)	82(47-194)	40(16-88)	56	48
Renal cancer	6	Advanced renal cancer, 1 (n=117); General renal cancer, 3 (n=318)	Europe, 3 (n=325); Asia, 3（n=439）	IHC, 4 (n=543); PCR, 2 (n=221)	764(436/328)	127(51-225)	73(32-110)	64	62
Lung cancer	7	Advanced non-small cell lung cancer, 2 (n=100); Non-small cell lung cancer, 3 (n=394), Lung cancer, 2 (n=233)	North america, 3 (n=254); Europe, 1 (n=61); Asia,3（n=412）	IHC, 5 (n=632); PCR, 1 (n=79); FACS, 1 (n=16)	727(295/432)	103(16-208)	42(5-117)	64	46
Melanoma	4	Uveal melanoma, 1 (n=25); Melanoma, 3 (n=143)	Europe, 4 (n=168)	IHC, 4 (n=168)	168(74/94)	42(25-71)	19(7-31)	59	50
Gynecologic cancer	8	Ovarian cancer, 6 (n=622); Vulvar cancer, 1 (n=30); Cervival cancer, 1 (n=174)	Europe, 2 (n=360); Asia,6（n=466）	IHC, 8 (n=826)	826(549/277)	103(30-241)	69(19-214)	56	66
Pancreatic cancer	2	None	Europe, 2 (n=320)	IHC, 2 (n=320)	320(254/66)	160(71-249)	127(39-215)	63	ND
Prostate cancer	2	None	Asia, 2 (n=109)	IHC, 2 (n=109)	109(54/55)	55(52-57)	27(18-36)	69	39
Liver cancer	2	None	North america, 1 (n=75); Asia, 1（n=181）	IHC, 2 (n=256)	256(138/118)	128(75-181)	69(47-91)	51	44
Sarcoma	2	Soft tissue sarcoma, 1 (n=112); Osteosarcoma, 1 (n=56)	Asia, 2 (n=168)	IHC, 1 (n=56); PCR, 1(n=112)	168(97/71)	84(56-112)	49(39-58)	18	38
Gallbladder cancer	1	None	Asia, 1 (n=72)	IHC, 1 (n=72)	72(50/22)	72	50	60	30
**Total**	**85**		**North america, 18 (n=1796) Europe, 25 (n=4493); Asia, 41（n=4639） South america, 1 (n=104)**	**IHC, 65 (n=9101); PCR, 8 (n=641); WB, 7 (n=786); FACS, 5 (n=504)**	**11032(5791/5241)**	**130(16-1382)**	**68(5-969)**	**58**	**53**

### CXCR4 and PFS

Forty-seven studies, with a total of 5,592 patients, included data on progress free survival in 12 types of cancer 8, 10, 12, 14-27, 31-33, 35, 38-40, 43, 45-48, 53, 56, 63-66, 70, 73, 75, 77-80, 82, 83, 85, 88, 89]. Of all the participants, 2583 (46%) were CXCR4 over-expression. Out of a maximum 9-point score in quality examination, 26 studies (55%) were at low risk of bias, the rest 21 (45%) were at high risk of bias. 20 studies (43%) had PFS adjust to covariates. Geographically, 14 studies (30%) including 1617 participants were conducted in the US and Canada, 15 studies (32%) including 2176 participants in Europe, and 18 studies (38%) including 1799 participants in Asia.

**Figure 2:**
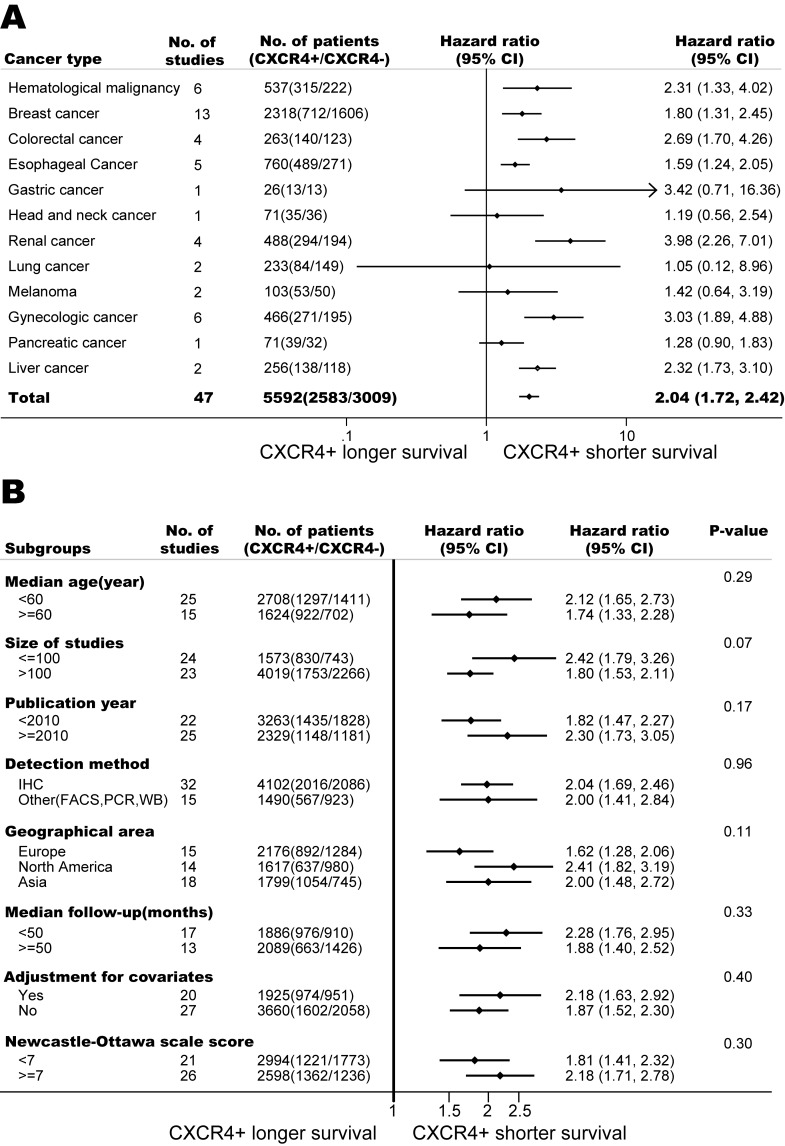
Association of CXCR4 over-expression and progress free survival (PFS) (A) Forest plot shows CXCR4 over-expression and PFS in 12 types of cancers. (B) CXCR4 over-expression is associated with worse PFS among cancer patients according to various characteristics. CI, confidence interval. Pooled estimates are based on random effects meta-analysis. Horizontal line represents 95% CI.

As shown in Figure 2A, CXCR4 over-expression was statistically associated with a poor PFS (HR=2.04, 95% CI, 1.72-2.42) when including all 47 studies; however, there was significant heterogeneity (I^2^ =71%, p<0.01). Meta-influence analysis did not suggest undue influence of any single study. Although five studies appeared to be outliers [8, 25, 63, 66, 77], we did not find clinical heterogeneity justifying their exclusion. Subsidiary analyses were carried out in the analysis of PFS (Figure 2B). In the eight predefined subgroup analyses, the prognostic effects were similar between the subgroups by line of risk of bias (Newcastle-Ottwa scale scores), levels of adjustment, median follow-up period, geographical area, detection methods, publication year, size of studies and age. These results might indicate that the predictive ability of CXCR4 over-expression is independent of other clinical and pathological factors for the survival of cancer patients.

**Figure 3:**
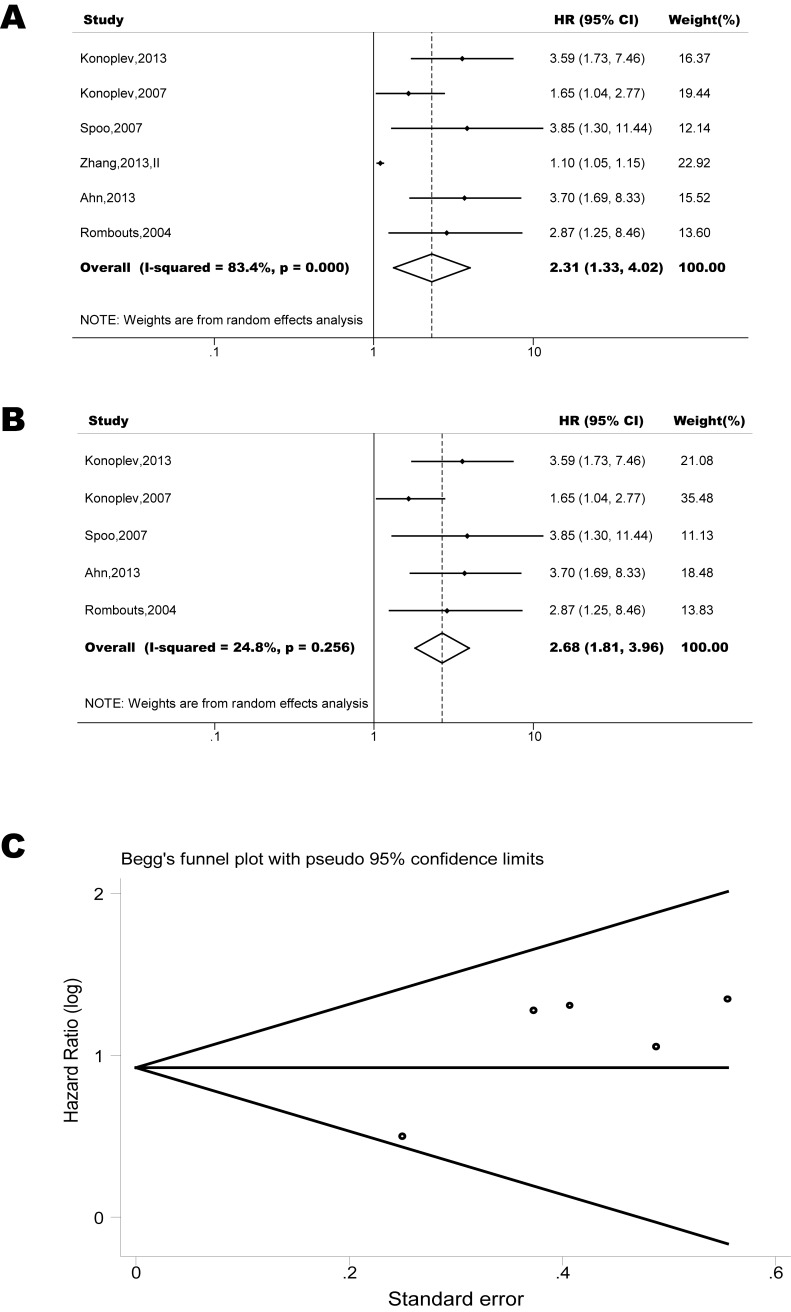
Forest plots show association between C-X-C chemokine receptor type 4 (CXCR4) over-expression and progression free survival (PFS) in hematological malignancy Hazard ratios for each trial are represented by squares; the horizontal line crossing the square represents the 95% confidence interval. The diamonds represent the estimated pooled effect using the Mantel-Haenszel random-effect model. (A) Summary for all six trials, the estimates is 1.12(1.07-1.17) using fixed effects model. (B) Excluding the only study focused on myelodysplastic syndrome (Zhang, 2012) yield results without significant heterogeneity. The estimate is 2.62(1.82-3.48) using fixed effects model. (C) Funnel plots showing association of CXCR4 and PFS in hematological malignancy. Visual inspection of the Begg funnel plot did not identify substantial asymmetry.

**Figure 4:**
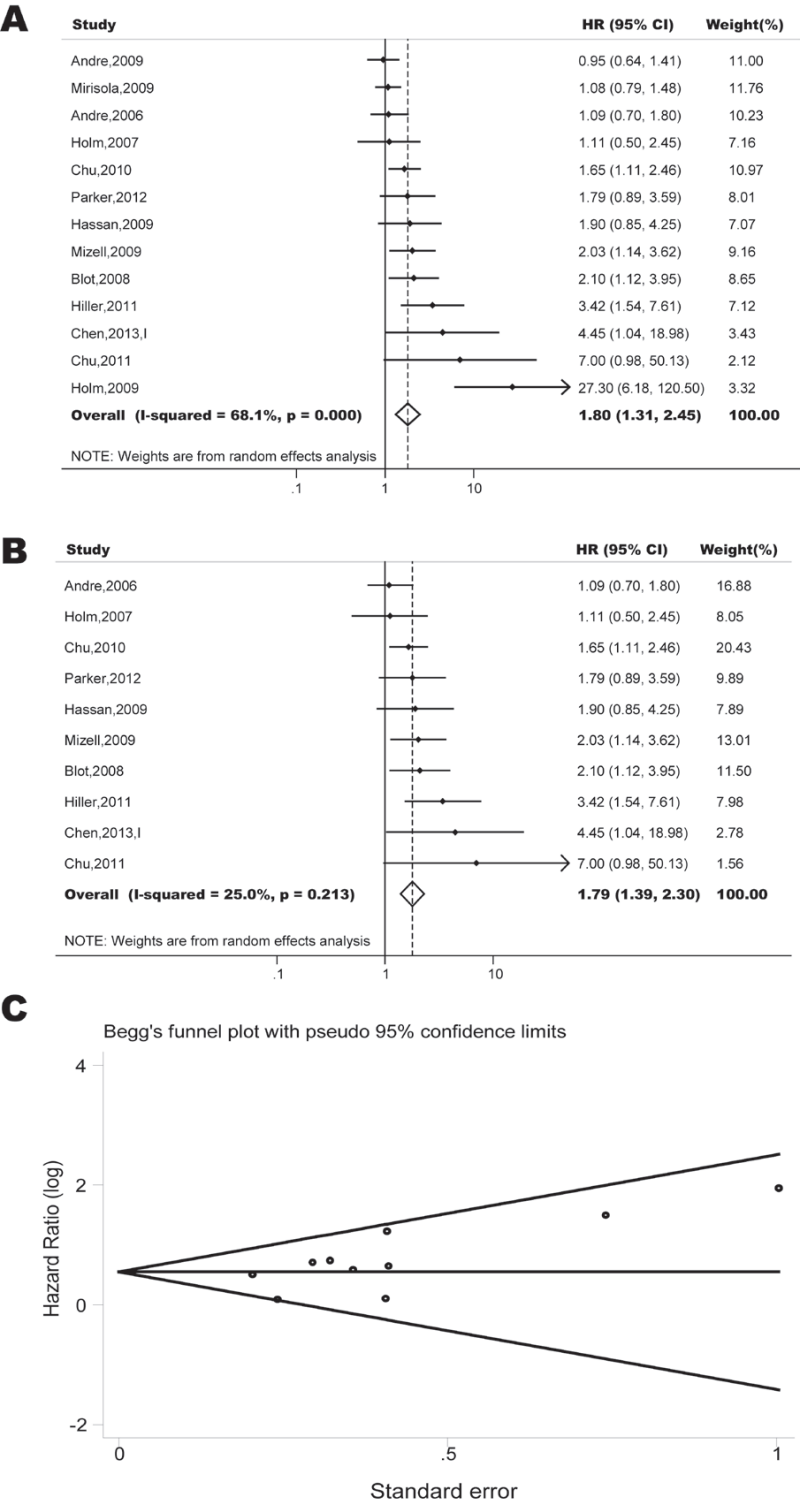
Forest plots show association between CXCR4 over-expression and PFS in breast cancer (A) Summary for all thirteen trials, the estimates is 1.44(1.23-1.69) using fixed effects model. (B) Although three studies appeared to be outliers (Andre, 2009; Mirisola, 2009; Holm, 2009), we did not find clinical heterogeneity justifying their exclusion. Excluding three studies yield similar results but without significant heterogeneity. The estimate is 1.75(1.41-2.17) using fixed effects model. (C) Funnel plots showing association of CXCR4 and PFS in breast cancer. Visual inspection of the Begg funnel plot did not identify substantial asymmetry.

**Figure 5:**
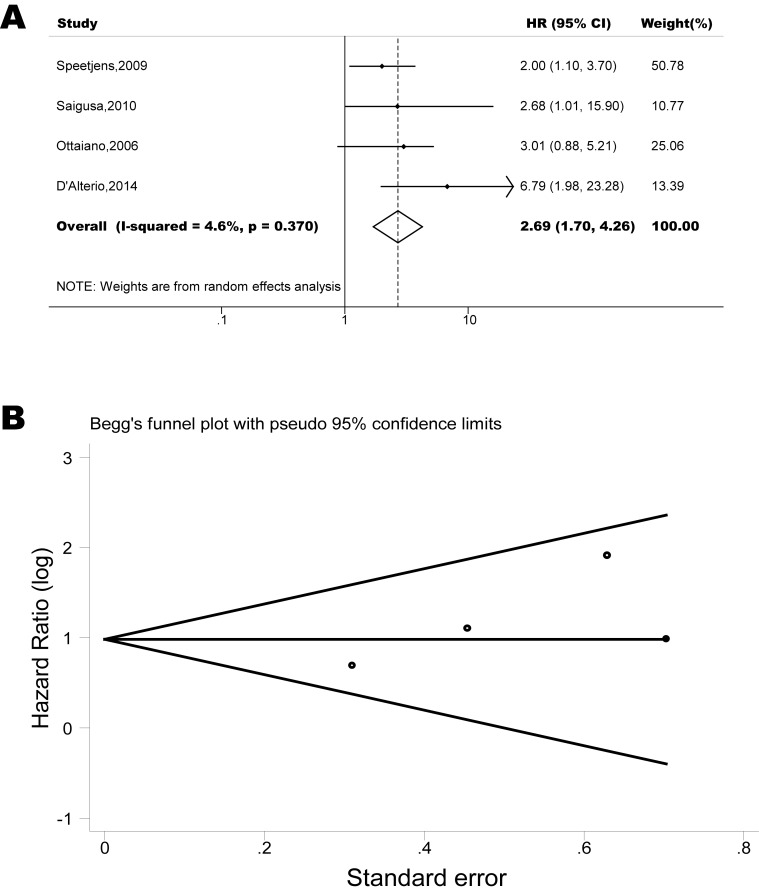
Forest plots show association between CXCR4 over-expression and PFS in colorectal cancer (A) Summary for all four trials, the estimates is 2.67(1.71-4.13) using fixed effects model. (B) Funnel plots showing association of CXCR4 and PFS in colorectal cancer. Visual inspection of the Begg funnel plot did not identify substantial asymmetry.

**Figure 6:**
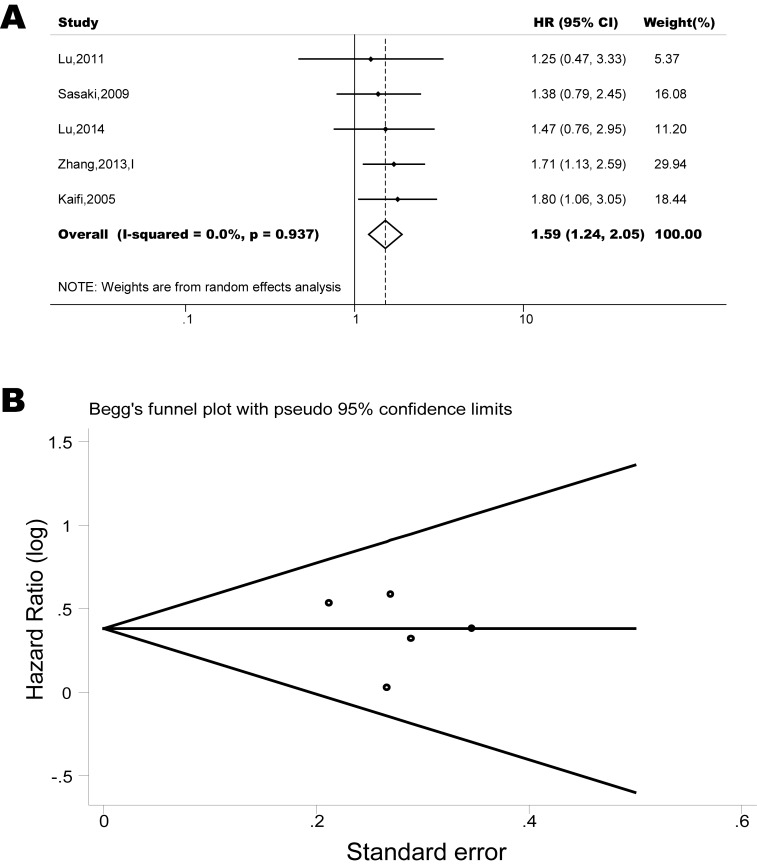
Forest plots show association between CXCR4 over-expression and PFS in esophageal cancer (A) Summary for all six trials, the estimates is 1.59(1.24-2.05) using fixed effects model. (B) Funnel plots showing association of CXCR4 and PFS in esophageal cancer. Visual inspection of the Begg funnel plot did not identify substantial asymmetry.

**Figure 7:**
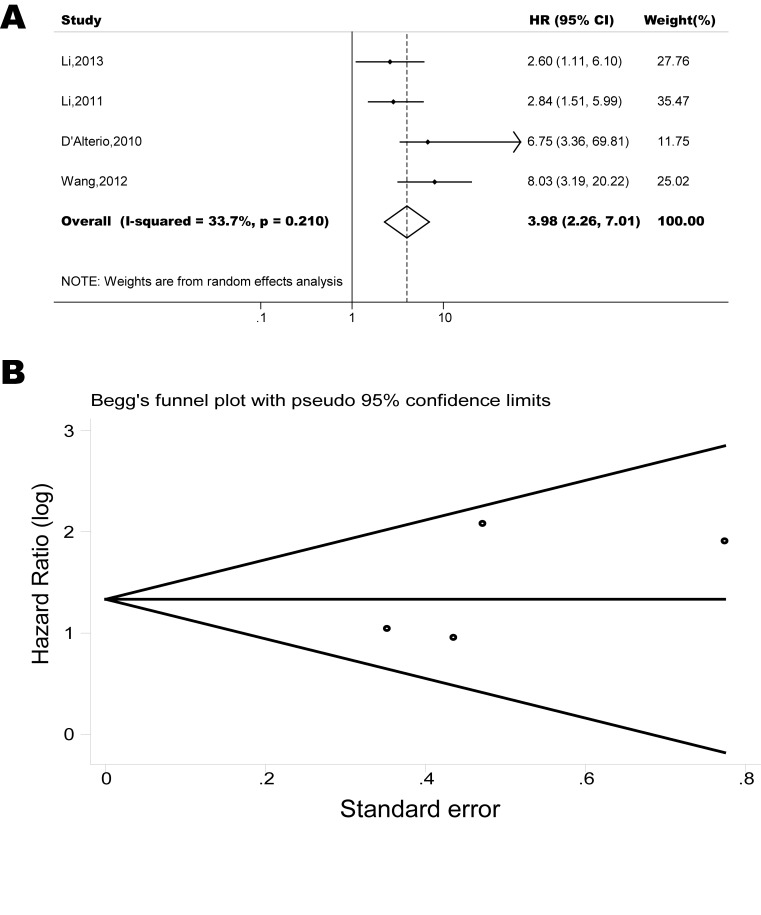
Forest plots show association between CXCR4 over-expression and PFS in renal cancer (A) Summary for all four trials, the estimates is 3.80(2.44-5.91) using fixed effects model. (B) Funnel plots showing association of CXCR4 and PFS in renal cancer. Visual inspection of the Begg funnel plot did not identify substantial asymmetry.

**Figure 8:**
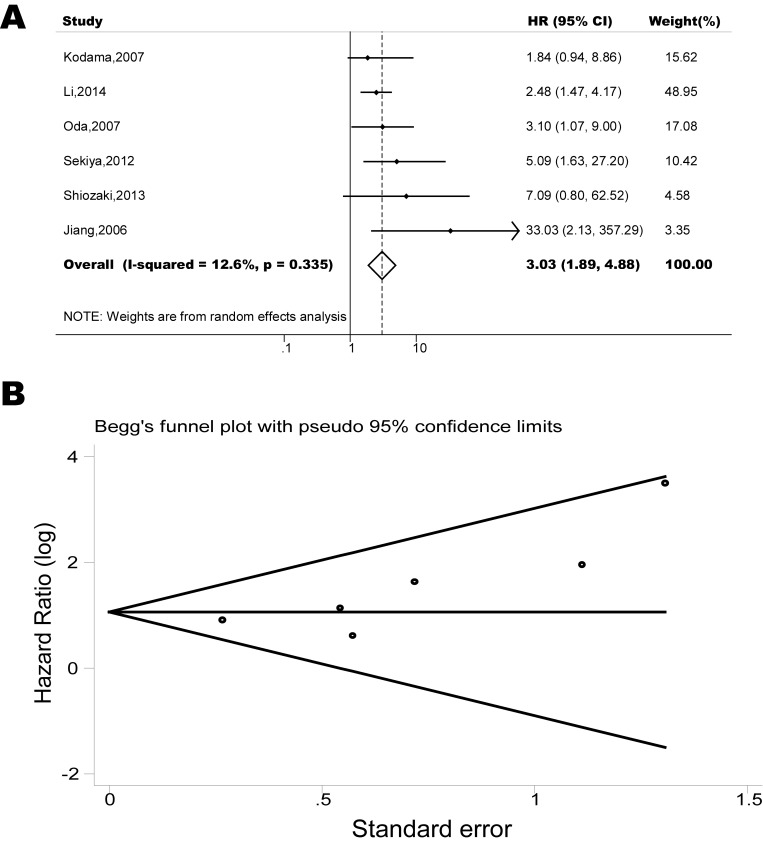
Forest plots show association between CXCR4 over-expression and PFS in gynecologic cancer (A) Summary for all six trials, the estimates is 2.89(1.93-4.32) using fixed effects model. (B) Funnel plots showing association of CXCR4 and PFS in gynecologic cancer. Visual inspection of the Begg funnel plot did not identify substantial asymmetry.

The pooled model showed a significantly shorter PFS with CXCR4 over-expression patients in hematological malignancy (6 studies, 537 patients, HR=2.31, 95% CI, 1.33-4.02, Figure 3) [12, 14-18], breast cancer (13 studies, 2318 patients, HR=1.80, 95% CI, 1.31-2.45, Figure 4) [10, 19-27, 30, 32, 33], colorectal cancer (4 studies, 263 patients, HR=2.69, 95% CI, 1.70-4.26, Figure 5) [35, 38-40], esophageal cancer (5 studies, 760 patients, HR=1.59, 95% CI, 1.24-2.05, Figure 6) [43, 45-48], renal cancer (4 studies, 488 patients, HR=3.98, 95% CI, 2.26-7.01, Figure 7) [63-66], gynecologic cancer (6 studies, 466 patients, HR=3.03, 95% CI, 1.89-4.88, Figure 8) [77-80, 82, 83] and liver cancer (2 studies, 256 patients, HR=2.32, 95% CI, 1.73-3.10) [88, 89]. Based on the available data, the associations between CXCR4 over-expression and PFS were inconclusive in gastric cancer (1 studies, 26 patients, HR=3.42, 95% CI, 0.71-16.36) [53], head and neck cancer (1 studies, 71 patients, HR=1.19, 95% CI, 0.56-2.54) [56], lung cancer (2 studies, 233 patients, HR=1.05, 95% CI, 0.12-8.96) [8, 70], melanoma (2 studies, 103 patients, HR=1.42, 95% CI, 0.64-3.19) [73, 75], pancreatic cancer (1 studies, 71 patients, HR=1.28, 95% CI, 0.90-1.83) [85]. Because of the small sample sizes of these five types of cancers, meaningful analysis of the role of CXCR4 on outcome in patients with these cancers were not possible.

### CXCR4 and OS

Seventy-nine studies, with a total of 10,506 patients, included data on overall survival in 15 types of cancer. Of all the participants, 5507 (52%) were CXCR4 over-expression. The overall quality of the included studies was examined, 40 studies (51%) were at low risk of bias, and the rest 39 studies (49%) were at high risk of bias. Twenty-seven studies (34%) had their outcomes adjust to different covariates. Geographically, 17 studies (22%) including 1642 participants were conducted in the US and Canada, 1 study (1%) including 104 participants were conducted in South America, 21 studies (27%) including 4151 participants in Europe, and 40 studies (50%) including 4609 participants in Asia.

**Figure 9:**
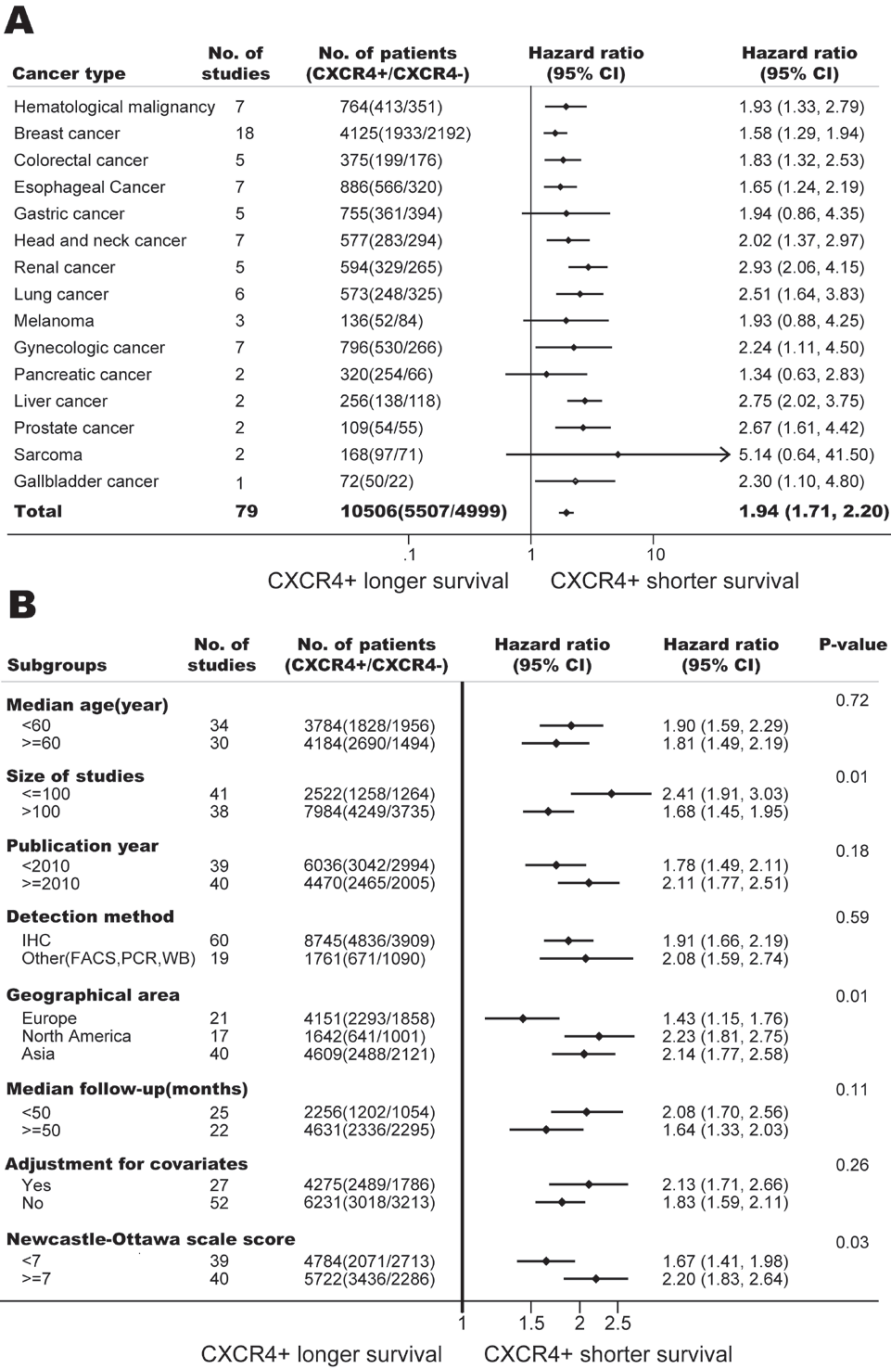
Association of CXCR4 over-expression and overall survival (OS) (A) Forest plot shows CXCR4 over-expression and OS in 15 types of cancers. (B) CXCR4 over-expression is associated with worse OS among cancer patients according to various characteristics.

As shown in Figure 9A, CXCR4 over-expression was statistically associated with a poor OS (HR=1.94, 95% CI, 1.71-2.20) when including all 79 studies [9-34, 36, 37, 39-62, 64-69, 71, 72, 74-92] ; however, there was significant heterogeneity (I^2^ =73%, p<0.01). Meta-influence analysis did not suggest undue influence of any single study. In the eight predefined subgroup analyses of OS (Figure 9B), the prognostic effects were similar between the subgroups by line of levels of adjustment, age, follow-up period, detection methods and publication year. However, the subgroup results by risk of bias (Newcastle-Ottwa scale scores); size of studies and geographical area appeared to be discordant. These may partly explain the substantial heterogeneities in our meta-analysis.

**Figure 10:**
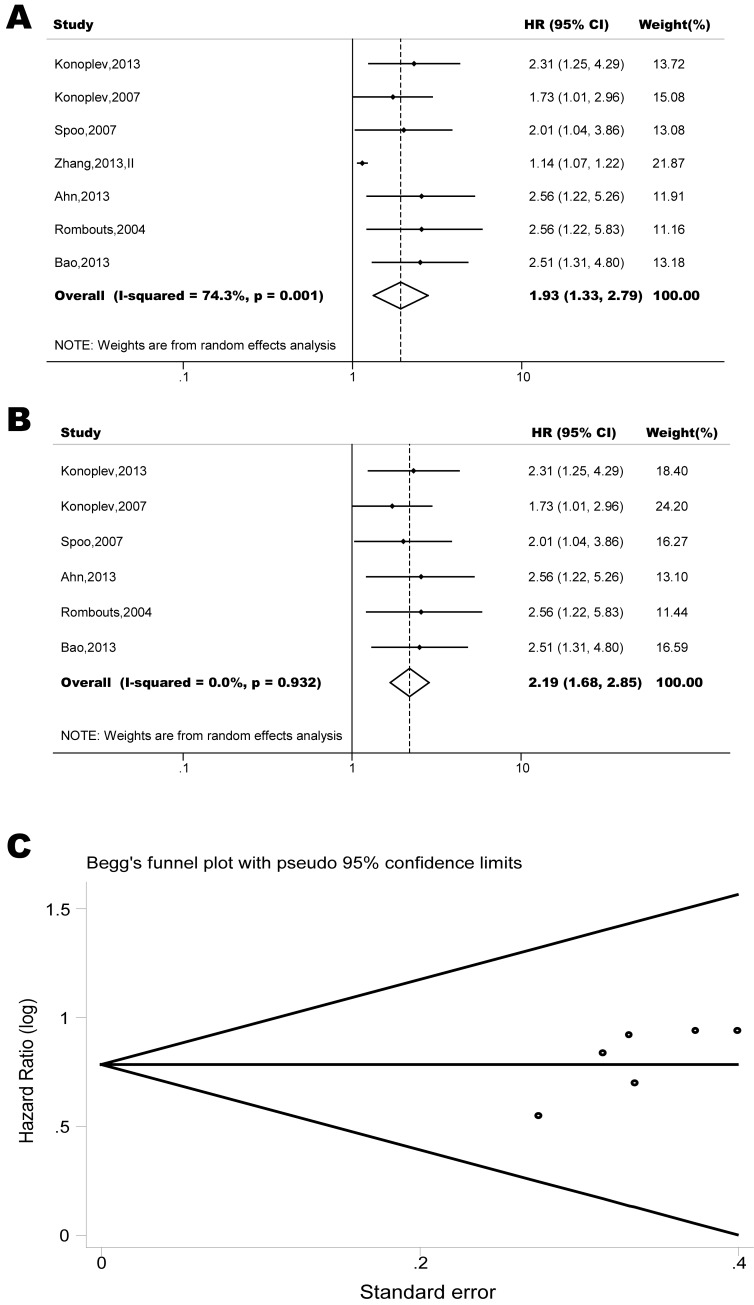
Forest plots show association between CXCR4 over-expression and overall survival (OS) in hematological malignancy (A) Summary for all seven trials, the estimates is 1.18(1.11-1.26) using fixed effects model. (B) Excluding the only study focused on myelodysplastic syndrome (Zhang, 2012) yield results without significant heterogeneity. The estimate is 2.19(1.68-2.85) using fixed effects model. (C) Funnel plots showing association of CXCR4 and OS in hematological malignancy. Visual inspection of the Begg funnel plot did not identify substantial asymmetry.

**Figure 11:**
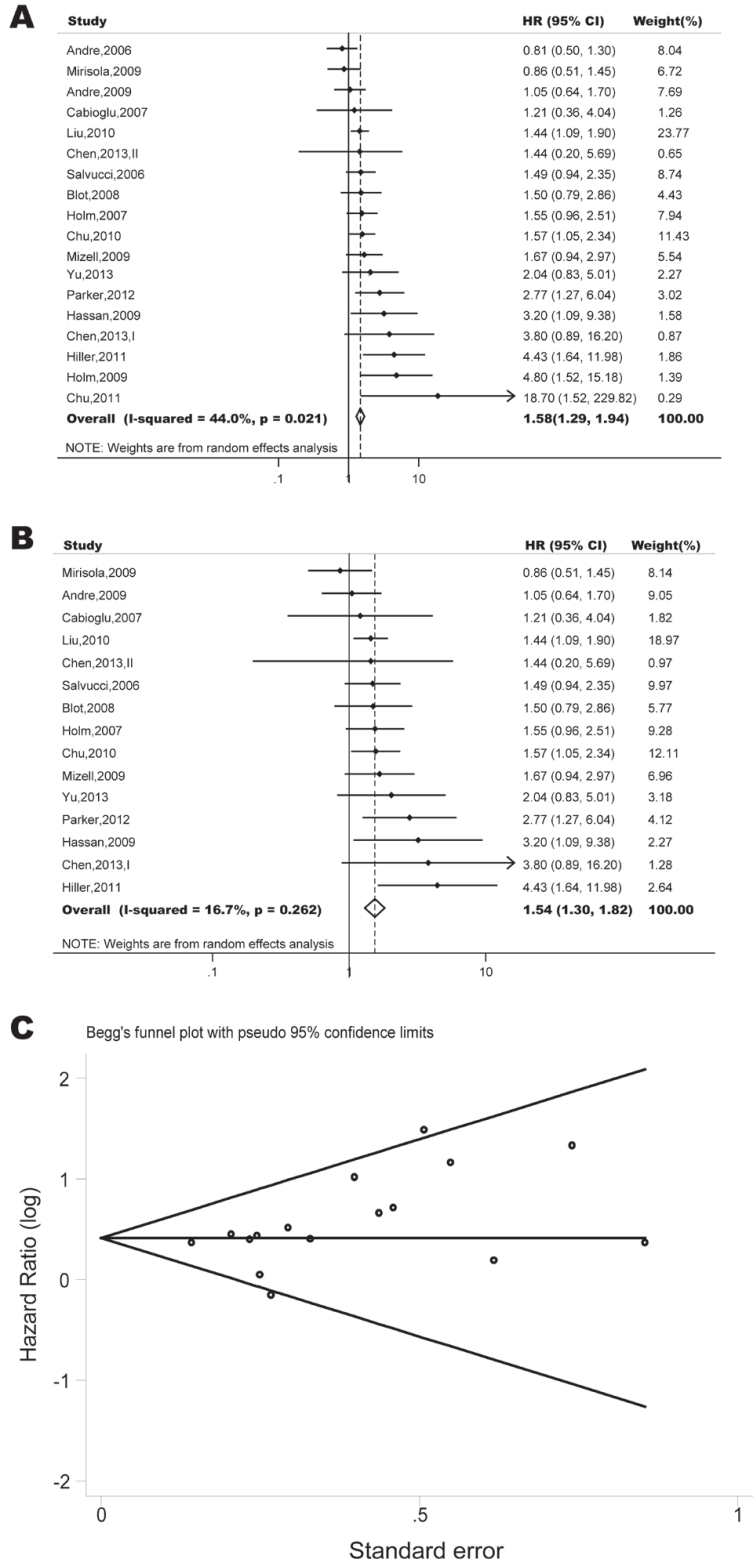
Forest plots show association between CXCR4 over-expression and OS in breast cancer (A) Summary for all nineteen trials, the estimates is 1.47(1.29-1.69) using fixed effects model. (B) Although three studies appeared to be outliers (Andre, 2006; Chu, 2011; Holm, 2009), we did not find clinical heterogeneity justifying their exclusion. Excluding three studies yield similar results but without significant heterogeneity. The estimate is 1.52(1.31-1.75) using fixed effects model. (C) Funnel plots showing association of CXCR4 and OS in breast cancer. Visual inspection of the Begg funnel plot did not identify substantial asymmetry.

**Figure 12:**
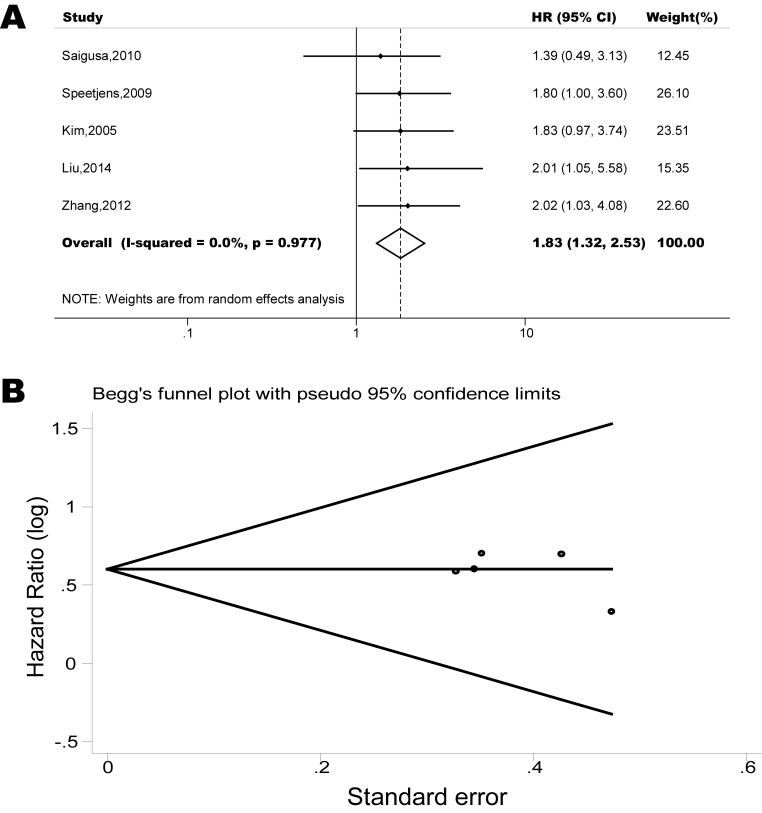
Forest plots show association between CXCR4 over-expression and OS in colorectal cancer (A) Summary for all five trials, the estimates is 1.83(1.32-2.53) using fixed effects model. (B) Funnel plots showing association of CXCR4 and OS in colorectal cancer. Visual inspection of the Begg funnel plot did not identify substantial asymmetry.

**Figure 13:**
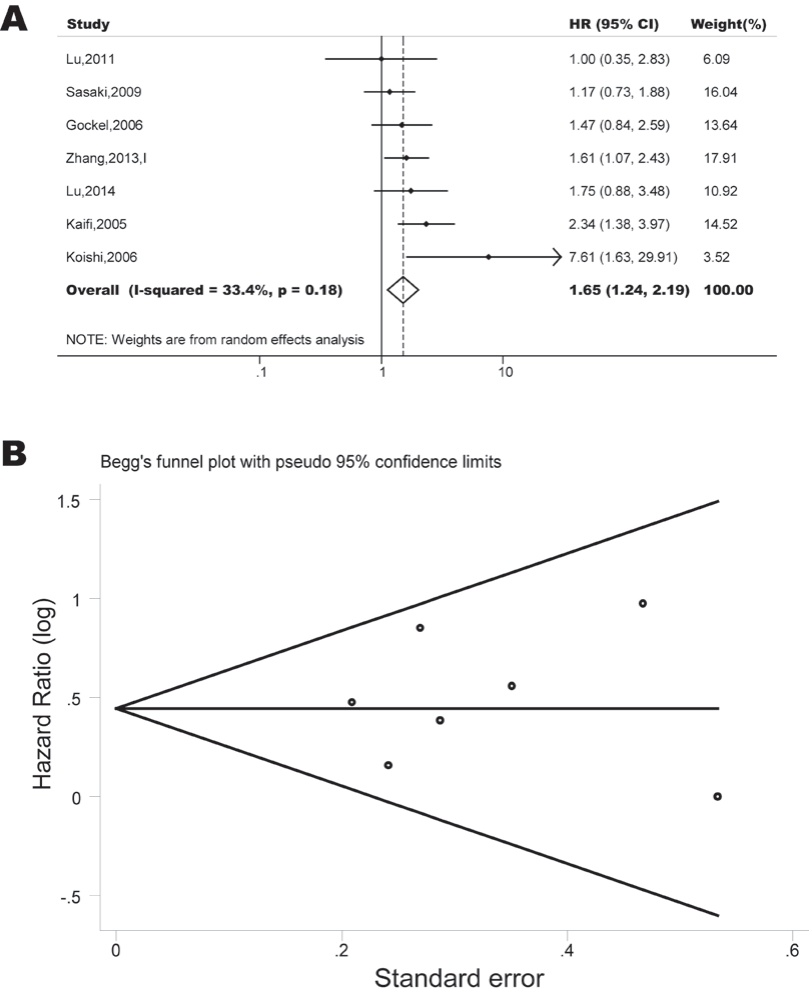
Forest plots show association between CXCR4 over-expression and OS in esophageal cancer (A) Summary for all eight trials, the estimates is 1.63(1.22-2.09) using fixed effects model. (B) Funnel plots showing association of CXCR4 and OS in esophageal cancer. Visual inspection of the Begg funnel plot did not identify substantial asymmetry.

**Figure 14:**
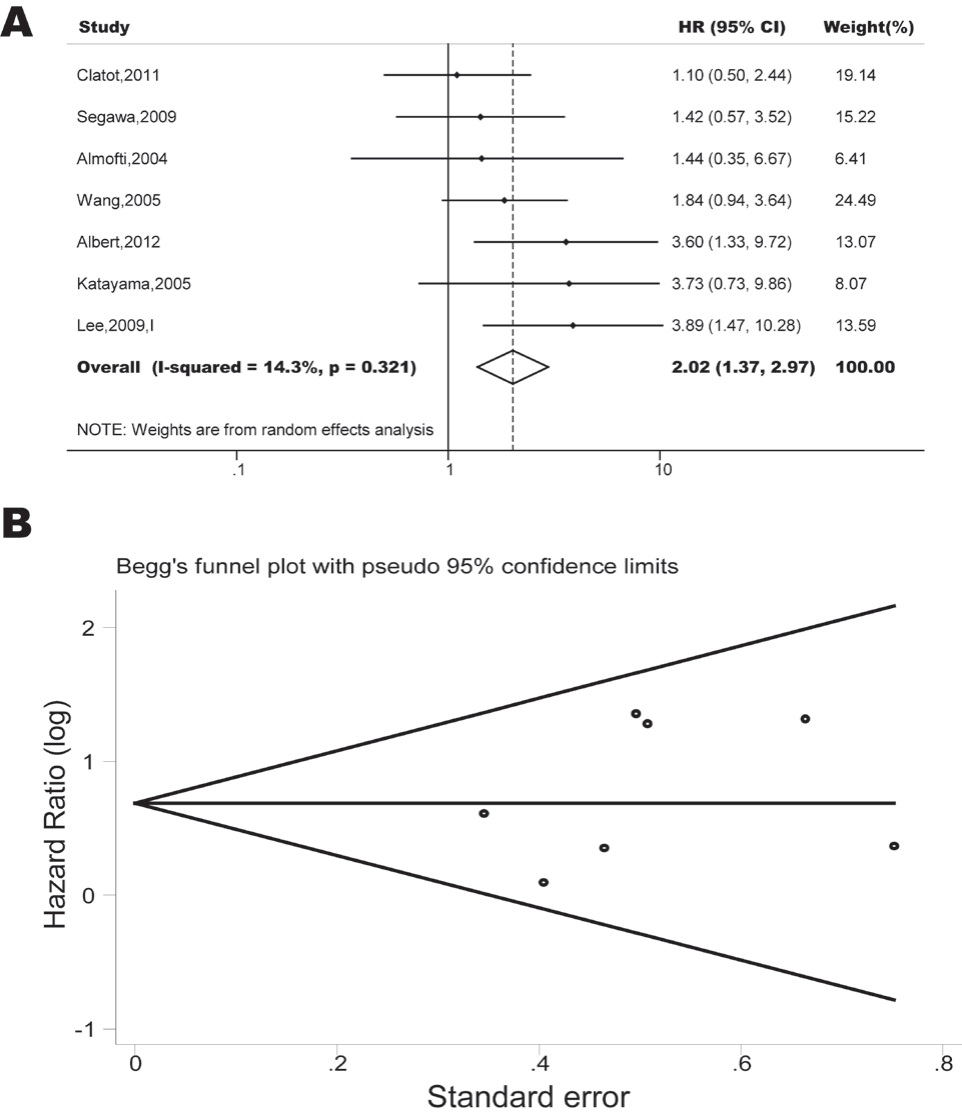
Forest plots show association between CXCR4 over-expression and OS in head and neck cancer (A) Summary for all seven trials, the estimates is 2.00(1.40-2.81) using fixed effects model. (B) Funnel plots showing association of CXCR4 and OS in head and neck cancer. Visual inspection of the Begg funnel plot did not identify substantial asymmetry.

**Figure 15:**
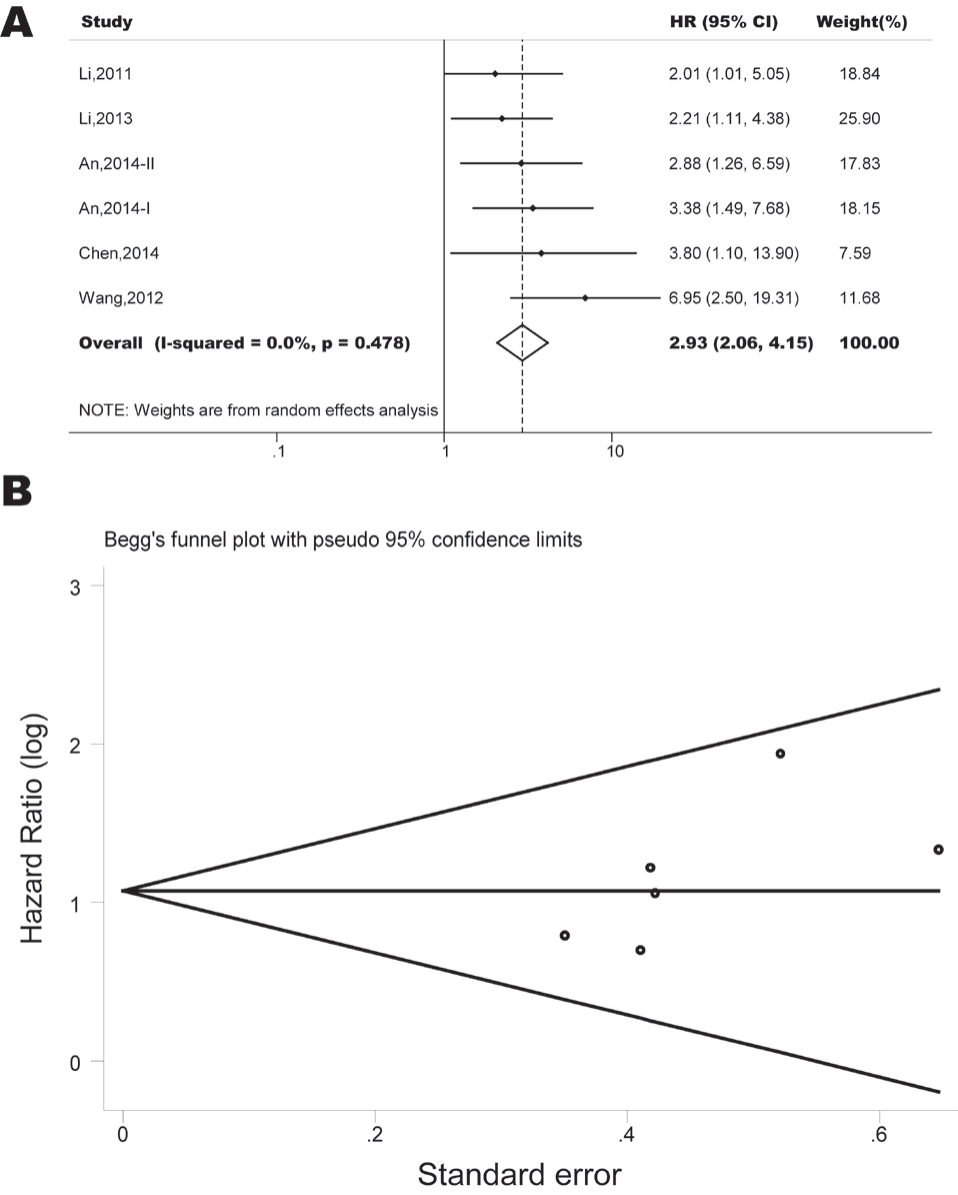
Forest plots show association between CXCR4 over-expression and OS in renal cancer (A) Summary for all six trials, the estimates is 2.93(2.06-4.15) using fixed effects model. (B) Funnel plots showing association of CXCR4 and OS in renal cancer. Visual inspection of the Begg funnel plot did not identify substantial asymmetry.

**Figure 16:**
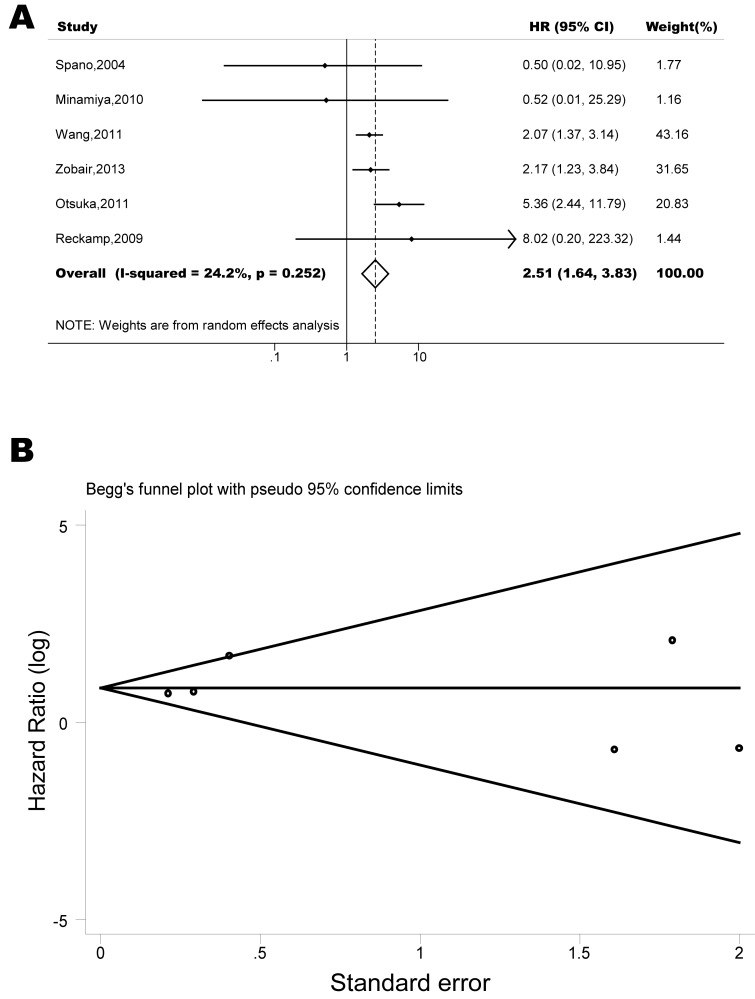
Forest plots show association between CXCR4 over-expression and OS in lung cancer (A) Summary for all six trials, the estimates is 2.40(1.76-3.25) using fixed effects model. (B) Funnel plots showing association of CXCR4 and OS in lung cancer. Visual inspection of the Begg funnel plot did not identify substantial asymmetry.

**Figure 17:**
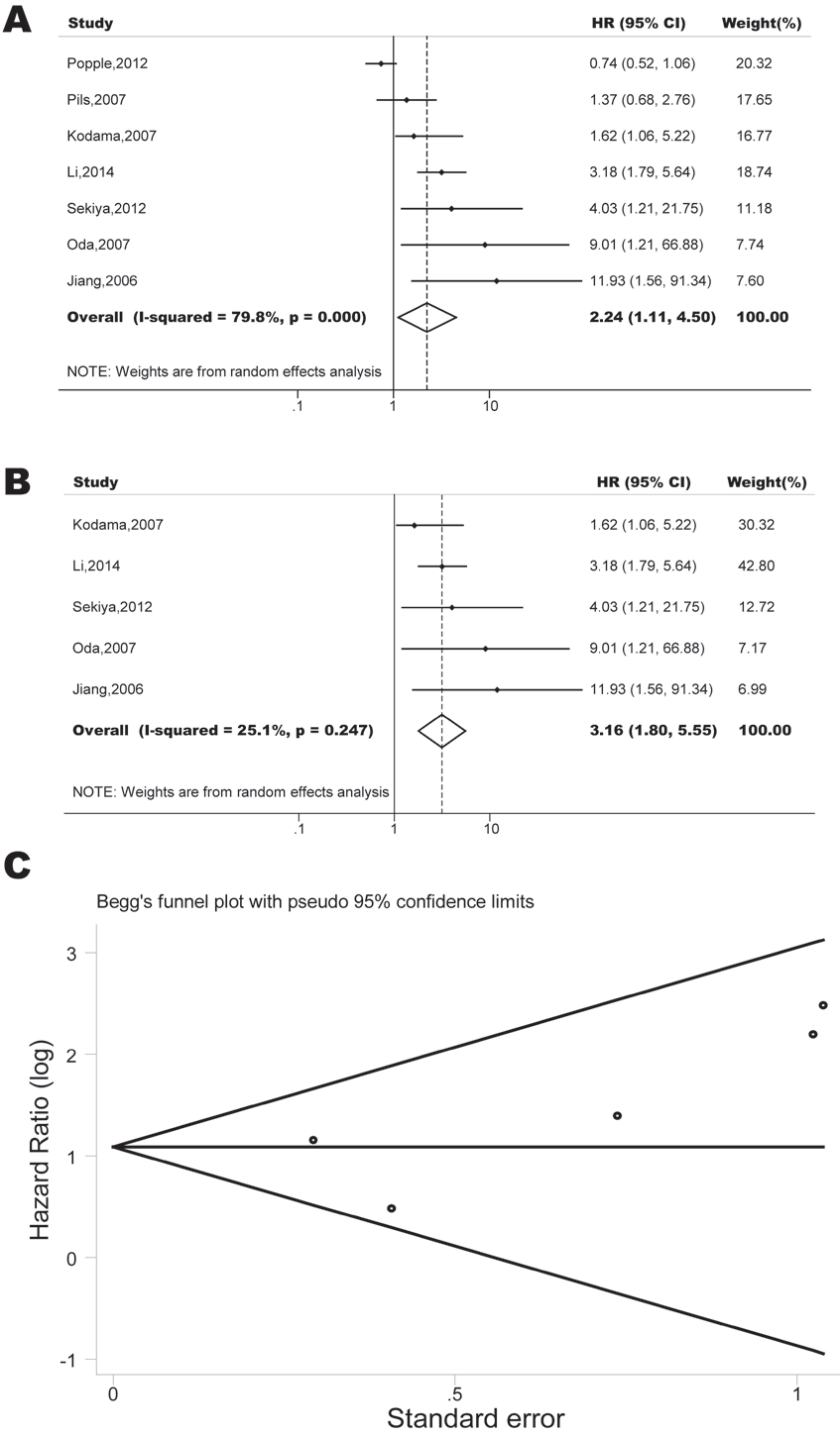
Forest plots show association between CXCR4 over-expression and OS in gynecologic cancer (A) Summary for all seven trials, the estimates is 1.32(1.03-1.71) using fixed effects model. (B) Excluding two studies (Popple, 2012; Pils, 2007) yield results without significant heterogeneity. The estimate is 3.00(1.85-4.55) using fixed effects model. (C) Funnel plots showing association of CXCR4 and OS in gynecologic cancer. Visual inspection of the Begg funnel plot did not identify substantial asymmetry.

**Figure 18:**
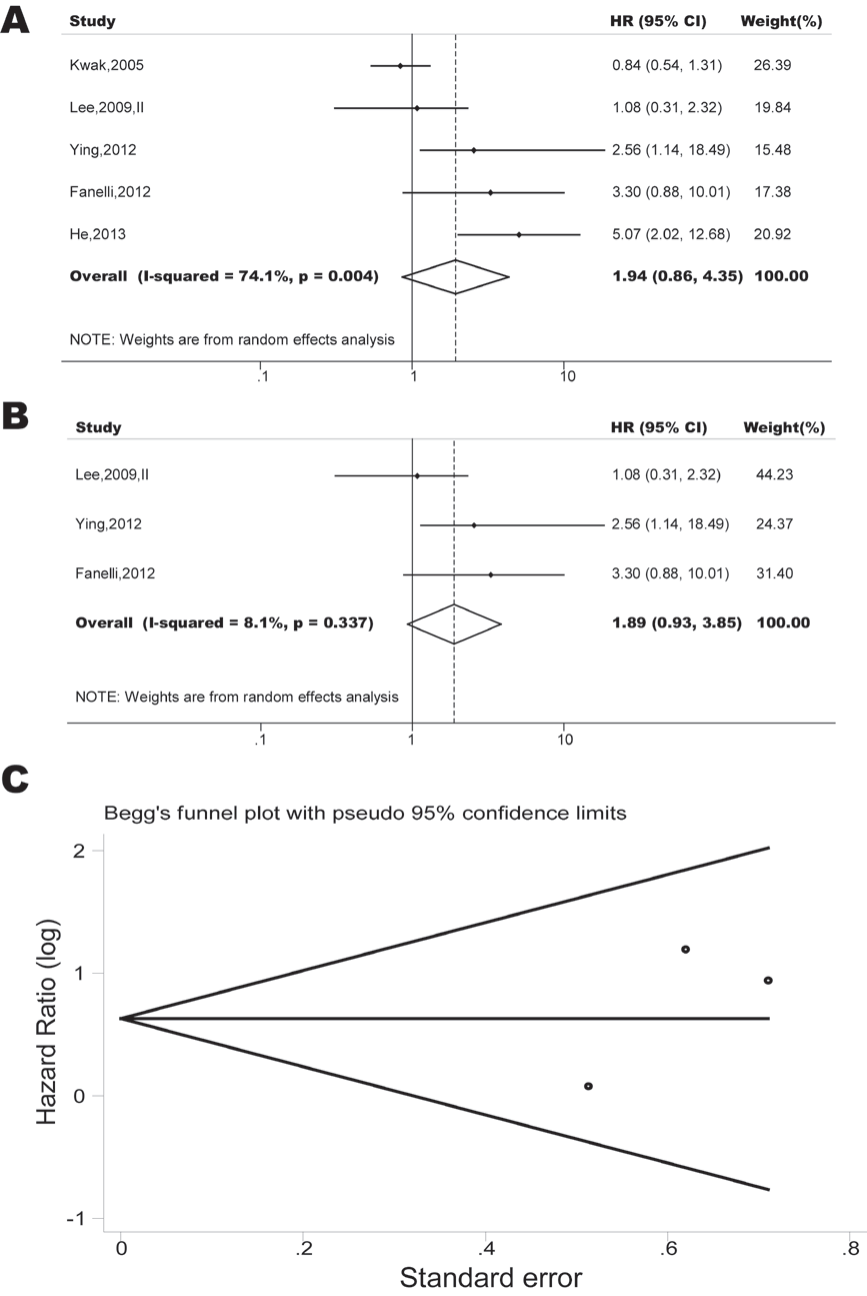
Forest plots show association between CXCR4 over-expression and OS in gastric cancer (A) Summary for all five trials, the estimates is 1.33(0.94-1.87) using fixed effects model. (B) Although two studies appeared to be outliers (Koishi, 2006; Sasaki, 2009), we did not find clinical heterogeneity justifying their exclusion. Excluding two studies yield results without significant heterogeneity. The estimate is 1.88(0.95-3.70) using fixed effects model. (C) Funnel plots showing association of CXCR4 and OS in gastric cancer. Visual inspection of the Begg funnel plot did not identify substantial asymmetry.

**Figure 19:**
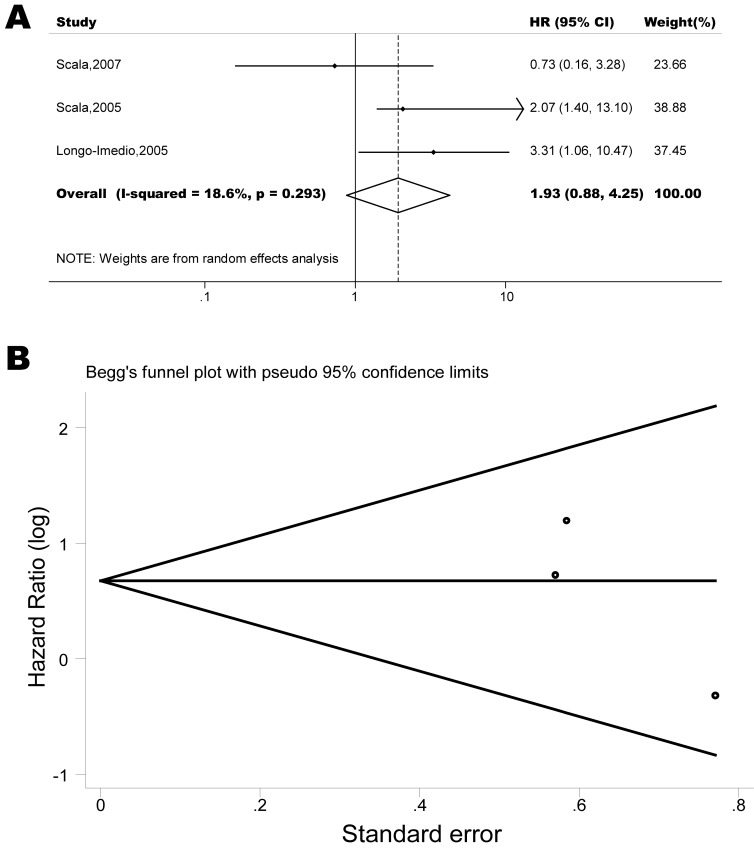
Forest plots show association between CXCR4 over-expression and OS in melanoma (A) Summary for all three trials, the estimates is 1.96(0.97-3.99) using fixed effects model. (B) Funnel plots showing association of CXCR4 and OS in melanoma. Visual inspection of the Begg funnel plot did not identify substantial asymmetry.

The pooled model showed a significantly shorter OS with CXCR4 over-expression patients in hematological malignancy (7 studies, 764 patients, HR=1.93, 95% CI, 1.33-2.79, Figure 10) [12-18], breast cancer (18 studies, 4125 patients, HR=1.58, 95% CI, 1.29-1.94, Figure 11) [10, 11, 19-34], colorectal cancer (5 studies, 375 patients, HR=1.83, 95% CI, 1.32-2.53, Figure 12) [36, 37, 39-41], esophageal cancer (7 studies, 886 patients, HR=1.65, 95% CI, 1.24-2.19, Figure 13) [42-48], head and neck cancer (7 studies, 577 patients, HR=2.02, 95% CI, 1.37-2.97, Figure 14) [54-60], renal cancer (5 studies, 594 patients, HR=2.93, 95% CI, 2.06-4.15, Figure 15) [61, 62, 64-66], lung cancer (6 studies, 573 patients, HR=2.51, 95% CI, 1.64-3.83, Figure 16) [8, 67-69, 71, 72], gynecologic cancer (7 studies, 796 patients, HR=2.24, 95% CI, 1.11-4.50, Figure 17) [9, 77-82], liver cancer (2 studies, 256 patients, HR=2.75, 95% CI, 2.02-3.75) [88, 89], prostate cancer (2 studies, 109 patients, HR=2.67, 95% CI, 1.61-4.42) [86, 87] and gallbladder cancer (1 studies, 72 patients, HR=2.30, 95% CI, 1.10-4.80) [92]. Based on the available data, the associations between CXCR4 over-expression and PFS were inconclusive in gastric cancer (5 studies, 755 patients, HR=1.94, 95% CI, 0.86-4.35, Figure 18) [49-53], melanoma (3 studies, 136 patients, HR=1.93, 95% CI, 0.88-4.25, Figure 19) [74-76], pancreatic cancer (2 studies, 320 patients, HR=1.34, 95% CI, 0.63-2.83) [84, 85] and sarcoma (2 studies, 168 patients, HR=5.14, 95% CI, 0.64-41.50) [90, 91]. The insignificant associations between these subtypes of cancer and clinical outcome might be due to the limited available data.

## DISCUSSION

CXCR4 has been implicated in the etiology of a substantial number of tumors because this receptor is thought to play a key role in chemotaxis, invasion, angiogenesis, metastasis and proliferation. Many agents against CXCR4 are currently under development [2]. However, there still remain unanswered questions about the direction and magnitude of effect of CXCR4 on outcome and whether the outcome is consistent among different subgroups. Here, we report a systematic review of 11,032 patients included in 85 different studies. Our study shows that the expression of CXCR4 is a significant and independent biomarker of worse prognosis in cancer. This result may suggest that the development of strategies against CXCR4 could be a reasonable therapeutic approach.

### CXCR4 as an independent prognostic biomarker in cancer

CXCR4 is expressed in various different tumor types and has been considered the most widely expressed chemokine receptor in most cancers. In current meta-analysis, we revealed that CXCR4 over-expression were generally associated with poorer survival in most cancer. For certain types of cancer such as gastric cancer, sarcoma, pancreatic cancer and melanoma, these associations are inconclusive. We believe these insignificances were due to the small size of available studies. Further investigations were needed to clarify the role of CXCR4 as a biomarker for prognosis in these types of cancer.

At present, it is well accepted that CXCR4 over-expression is a risk factor of short survivals in certain types of cancer. However, whether CXCR4 over-expression is independently associated with worse clinical outcome remains controversial. Results from our sensitivity analysis restrict to studies adjusted for established confounders such as age, gender and tumor stage, suggest that CXCR4 over-expression is probably an independent prognostic biomarker. Moreover, if CXCR4 was merely an early marker, it would be more likely to occur just the time of onset of cancer. In fact, the mean length of follow-up in primary studies ranged from 8 to 167 months. Such a large interval further supports the hypothesis that CXCR4 over-expression is an independent prognostic biomarker.

The underlying mechanisms involved in the association between CXCR4 over-expression and survivals are uncertain. One possible explanation is the “CXCL12/CXCR4 chemokine axis hypothesis” [93]. In the past decades, more and more evidence suggests the stroma contributes to the growth and invasion of tumors. In support of this notion, it was demonstrated that of CXCR4 may play a critical role as a chemoattractant in cancer development possibly at the level of the tumor niche [94]. The data reveals that both CXCL12 expression by fibroblasts and CXCR4 expression on cancer cells, within hypoxic areas of tumors, trigger tumor cell growth, motility and invasiveness. In addition, the stroma cells from specialized microenvironments actually modulate CXCR4 expression, which is responsible for tumorigenesis and tumor progression. Obviously, the tumor and stroma cell interactions is truly reciprocal; while stroma cells may support tumors, tumor cells in turn modulate the microenvironments. Hence, CXCR4 and CXCL12 form an important signaling axis between tumor cells and their microenvironment, with the interaction influencing the adhesion, migration and invasion of tumor cells, reflecting the strong association of CXCR4 with cancer metastasis.

### Strengths and implications of findings

Our meta-analyses have several important implications. First, the generalisability of our findings has been enhanced by the involvement of data from over 10,000 participants in 14 nations. Second, the association of CXCR4 over-expression with survivals persists and remains statistically significant based on various classification criteria. Third, all of the analyses were conducted by random-effects model and fixed-effects model, both models showed similar results, which indicated that the statistic results were robust.

In addition to being a prognostic biomarker, our results are of clinical relevance in view of the emergence of new drugs targeting CXCR4. Currently, only one CXCR4 antagonist, plerixafor is approved by FDA but several others are being investigated in clinical phase I/II trials (Table 2). Plerixafor in combination with granulocyte-colony stimulating factor (G-CSF) has been approved as mobiliser of haematopoietic CD34+ cells from the bone marrow to the circulation for patients with non-Hodgkin's lymphoma and multiple myeloma [2]. Also, it is currently involved in a number of clinical trials for the prevention of growth and metastasis of many different cancers. Besides the CXCR4 inhibitors listed in Table 2, CTCE-9908, one CXCL12 peptide analogue, was tested as mono-treatment in advanced solid tumor. In July 2005, FDA assigned orphan drug status to this drug for the treatment of osteosarcoma, but there was no further news on advancement of clinical trial although phase I/II trial had been completed in 2008 [95]. CXCR4 overexpression was associated with both PFS and OS in seven subtypes of cancers (hematological malignancy, breast cancer, colorectal cancer, esophageal cancer, renal cancer, gynecologic cancer, and liver cancer). Only three disease sites had more than five studies supporting the significance of CXCR4 in impacting both PFS and OS (breast cancer, gynecologic cancer and hematological malignancy). So these organ systems might be the potential targets in the future clinical interventions.

**Table 2:** Studies evaluating anti-human C-X-C chemokine receptor type 4 (CXCR4) therapeutic strategies in cancer (Clinical trials involving Plerixafor, the only CXCR4 antagonist approved by FDA, are not included in this table because of the limited space). BMS-936564 is monoclonal antibody against CXCR4. LY2510924, BL-8040, MSX-122, POL6326 and TG-0054 are CXCR4 antagonists

Study	Sponsor	Disease	Phase	Intervention(s)
NCT01359657	Bristol-Myers Squibb	Multiple Myeloma	Phase I	Arm A: BMS-936564 + Lenalidomide + Dexamethasone Arm B: BMS-936564 + Bortezomib + Dexamethasone
NCT01120457	Bristol-Myers Squibb	Acute Myelogenous Leukemia, Diffuse Large B-Cell Leukemia, Chronic Lymphocytic Leukemia, Follicular Lymphoma	Phase I	BMS-936564
NCT00591682	Metastatix Inc	Advanced Solid Tumors	Phase I	MSX-122
NCT01837095	Polyphor Ltd.	Metastatic Breast Cancer	Phase I	POL6326
NCT01010880	Biokine Therapeutics Ltd	Multiple Myeloma	Phase I/II	BL-8040, under the name BKT140
NCT01413568	Polyphor Ltd.	Acute Myelogenous Leukemia, Acute Lymphoblastic Leukemia, Chronic Myelogenous Leukemia, Non-Hodgkin's Lymphoma, Hodgkin's Disease, Chronic Lymphocytic Leukemia, Multiple Myeloma, Myelodysplastic Syndrome, Myeloproliferative Disorder	Phase I/II	POL6326
NCT02115672	Sheba Medical Center	Chronic Myeloid Leukemia	Phase I/II	BL-8040
NCT01018979	TaiGen Biotechnology Ltd.	Multiple Myeloma, Non-Hodgkin Lymphoma, Hodgkin Disease	Phase II	TG-0054
NCT01105403	Polyphor Ltd.	Multiple Myeloma	Phase II	POL6326
NCT01458288	TaiGen Biotechnology Ltd.	Multiple Myeloma, Non-Hodgkin Lymphoma, Hodgkin Disease	Phase II	TG-0054
NCT01838395	BioLineRx, Ltd.	Acute Myeloid Leukemia	Phase II	BL-8040 + Ara-C
NCT02104427	TaiGen Biotechnology Ltd.	Multiple Myeloma, Non-Hodgkin Lymphoma, Hodgkin Disease	Phase II	TG-0054 combined with G-CSF
NCT01439568	Eli Lilly and Company	Small Cell Lung Carcinoma	Phase II	Arm A: LY2510924 + Carboplatin + Etoposide Arm B: Carboplatin + Etoposide
NCT01391130	Eli Lilly and Company	Metastatic Renal Cell Carcinoma	Phase II	Arm A: LY2510924 + Sunitinib Arm B: Sunitinib

### Limitations of study

Despite of the strengths mentioned above, this meta-analysis also has some limitations. First, because this is a literature-based analysis, it is compromised by the potential for publication bias, whereby predominantly positive results were published, thus inflating our estimate for the association between CXCR4 and poor outcome. The languages of the published studies included in this meta-analysis were restricted to English. Other potentially eligible studies which met our inclusion criteria cannot be included. Second, this is a systematic review and meta-analysis of literatures, we were only able to extract population-level rather than individual patient level data. This reduced our ability to test for associations between variables in specific subgroups and also limited our ability to assess for sources of heterogeneity. Third, there was no accepted and validated method for assessment of CXCR4 expression. Therefore, there might be substantial heterogeneity, which might not be fully accounted for by our use of random-effects modeling. An internationally accepted and validated method for CXCR4 testing was needed. Fourth, the survival analysis was not performed by multivariate analyses in many studies reported; we calculated or estimated the HR from available data or Kaplan–Meier curves. Finally, there was marked heterogeneity in patient populations, clinical treatment method and follow-up of patients. Random-effects modeling and sensitivity analyses were conducted to address this heterogeneity, but these statistical methods may not be sufficient.

## MATERIALS AND METHODS

### Search strategy and selection of studies

This meta-analysis was carried out in accordance with the Preferred Reporting Items for Systematic Reviews and Meta-Analyses (PRISMA) statement [96]. Relevant studies published before June, 2014 (date last searched), were identified through electronic searches using PubMed and Embase. The following search terms were used: 1) cancer, tumor, neoplasm, carcinoma; 2) CXCR4, CXCR-4, C-X-C chemokine receptor type 4, CXC chemokine receptor 4, fusin, LESTR, HUMSTR, CD184, cluster of differentiation 184. Electronic searches were supplemented by scanning reference lists of articles identified for all relevant studies (including review articles), by hand searching of relevant journals and by correspondence with study investigators. In addition to full publications, original studies in the form of conference abstracts and letters were included to capture grey literature. Each study was assessed for inclusion by two or three reviewers independently and discrepancies within the reviewing pair were resolved via discussion.

All initially identified studies were screened of titles and/or abstracts; then full texts were retrieved for studies that satisfied all selection criteria. Studies were considered eligible if they met the following criteria: 1) the exposure of interest were cancer and CXCR4; 2) the outcome of interests were progression-free survival and overall survival; 3) hazard ratio (HR) and the corresponding 95% conﬁdence interval (CI) (or data sufficient to calculate them) were reported; and 4) exclusion of letters to the editor, reviews, and articles published in non-English language books or papers.

### Data Collection and extraction

We used a predesigned data abstraction form to extract relevant information. The following details were extracted: First author's full name; year of publication; country of origin; cancer type, median age at the time of diagnosis, median duration of follow-up, period of follow-up, method to detect CXCR4, total number of patients, number of CXCR4 over-expression patients and controls, method for CXCR4 assessment and cutoff for defining CXCR4 as over-expressed, reported adjusted factors and assessments of outcomes (HR and the corresponding 95% CI of PFS and/or OS). When the statistical variables were not given explicitly in an article, they were estimated from available data using methods reported by Tierney et al [97], or abstracted from other published reviews [98-100].

### Assessment of risk of bias

We used the Newcastle-Ottawa Scale to assess the risk of bias [101]. This scale uses a star system (with a maximum of nine stars) to evaluate a study in three domains: selection of participants, comparability of study groups, and the ascertainment of outcomes of interest. We judged studies that received a score of seven or more stars to be at low risk of bias, and those that scored less than seven to be at high risk of bias. This cutoff point was chosen according to the distribution of relative quality scores of all included studies.

### Statistical analysis

Homogeneity of HRs across the studies was tested by Q statistic (signiﬁcance level at p<0.05). The I^2^ statistic, a quantitative measure of inconsistency across studies [102], was also calculated. The combined risk estimates were computed by ﬁxed-effect models and random-effect models [103]. Fixed-effect models (P>0.1 and I^2^<50%) assume that the differences between the results of various studies are due to chance. Random-effect models (P < 0.1 or I^2^> 50%) assume that the results could genuinely differ between studies. When heterogeneity is present, the random-effect model is considered to be more appropriate than a fixed-effect model, resulting in wider intervals and a more conservative estimate of treatment effect.

Because characteristics of populations, ascertainment of different cancer subtype, and adjustments for confounding factors were not consistent between studies, we further conducted a sensitivity analysis by removing one or several studies to explore possible explanations for heterogeneity and to examine the inﬂuence of various exclusion criteria on the overall risk estimate.

Potential publication bias was assessed by visual inspection of the Begg funnel plots. We also performed the Begg rank correlation test at the p <0.10 level of signiﬁcance [104]. All analyses were performed using STATA version 12.0 (StataCorp LP, Texas). p<0.05 was considered statistically significant. All statistical tests were two-sided.

## CONCLUSIONS

Based on our review of 85 studies in over 11,000 patients with cancer, we show that over-expression of CXCR4 is associated with worse prognosis in terms of OS and PFS in different types of tumors, which suggests that the development of strategies against this receptor could be a reasonable therapeutic approach.

## SUPPLEMENTARY MATERIAL, FIGURES AND TABLES


