# Supplementary material for: CXCR4 over-expression and survival in cancer: A system review and meta-analysis
Source: Oncotarget. 2014 Dec 31;6(7):5022–40. doi: 10.18632/oncotarget.3217 (PMC4467131; doi:10.18632/oncotarget.3217)
Supplement: Supplementary file 1 [file oncotarget-06-5022-s001.pdf]

CXCR4 over-expression and survival in cancer: A system review and meta-analysis

Supplementary Material

Suppl.table.1: General characteristics of all studies included in the current meta-analysis.

IHC, immunohistochemistry; WB, western blot; RCR, polymerase chain reaction; FACS, fluorescence-activated cell sorting; ND, no data.

First author, year	Country	Disease	Detection method	No.of subjects (CXCR4+/CXCR4-)	Median age(range)	Median follow-up(months)	Period	Cutoff value detection
Chen,2014	Germany	Renal cell carcinoma	PCR	51(32/19)	ND	54	1992-2011	The martingale residual method
Minamiya,2010	Japan	Lung cancer	PCR	79(37/42)	62.3	ND	1998-2003	The threshold score for CXCR4 was 2.4 [(CXCR4 mRNA)/(GAPDH mRNA)]
Mirisola,2009	Germany	Breast cancer	IHC	100(47/53)	61	ND	2000-2002	Staining intensity score and percentage of positive cells, the threshold score was the median value
Lee,2009,I	Korea	Oral squamous cell carcinoma	IHC	74(45/29)	59.2(27-93)	ND	1995-2002	Staining intensity score. Positive: 1-3 staining point
Clatot,2011	France	Head and neck squamous cell carcinoma	PCR	71(35/36)	58(42-82)	ND	2004-2008	The threshold score for CXCR4 was 0.87 [(CXCR4 mRNA)/(PBGD mRNA)]
Albert,2012	France	Squamous cell carcinoma of tongue	IHC	47(23/24)	61(34-92)	48	2005-2008	The threshold immunohistochemical scores for CXCR4 was 120 based on the staining intensity
Konoplev,2013	US	Acute myeloid leukemia	IHC	101(66/35)	61(18-88)	8	2003-2008	Percentage of positive tumor cells. The threshold score for CXCR4 was 10%
Gockel,2006	Germany	Esophageal squamous cell and adenocarcinoma	IHC	102(64/38)	ND	ND	1999-2003	Staining intensity score,cutoff value was 1.5
Wang,2012	China	Renal cell carcinoma	IHC	97(60/37)	ND	ND	2002-2003	Percentage of positive tumor cells. The threshold score for CXCR4 was 30%
Zhang,2013,I	China	Esophageal squamous cell carcinoma	IHC	136(47/89)	62(35-90)	ND	2000-2002	Staining intensity score and percentage of positive cells, the threshold score was the median value
Speetjens,2009	Netherlands	Colorectal cancer	PCR,IHC	70(35/35)	ND	99	1990-2001	the threshold score was the median value
Konoplev,2007	US	Acute myeloid leukemia	IHC	122(70/52)	62(22-82)	18	1997-2003	Percentage of positive tumor cells. The threshold score for CXCR4 was 10%
Parker,2012	US	Node-positive breast cancer	WB	185(37/148)	59(41-85)	54	1987-2009	CXCR4 expression was defined as high (>7.5-fold) or low (<7.5-fold)
Oda,2009	Japan	Soft-tissue sarcoma	PCR	112(58/54)	ND	41.5	1988-2004	mRNA expression index = CXCR4 mRNA value/GAPDH mRNA value *1,000 AU,the threshold score was the median value
Andre,2006	France	Axillary node positive breast cancer	IHC	133(42/91)	50(32-63)	156	1972-1979	Staining intensity score,cutoff value was 3
Yopp,2012	US	Colorectal liver metastases	IHC	75(47/28)	ND	68	2002-2004	Percentage of positive tumor cells. The threshold score for CXCR4 was 10%
Katayama,2005	Japan	Head and neck squamous cell carcinoma	IHC	56(16/40)	ND	ND	1983-2003	Staining intensity score,score 1 to 3, CXCR4 positive; score 0, CXCR4 negative
Scala,2005	Italy	Malignant melanoma	IHC	71(31/40)	53	ND	1996-2003	Percentage of positive tumor cells. The threshold score for CXCR4 was 10%
Ottaiano,2006	Italy	Stage II-III colorectal cancer	IHC	72(56/16)	55(34-82)	23	2003-2004	Percentage of positive tumor cells. The threshold score for CXCR4 was 50%
Otsuka,2011	Canada	Stage IV non-small cell lung cancer	IHC	84(10/74)	69(32-96)	ND	2003-2006	Cutpoint from CXCR4 expression levels was calculated using log-rank test statistic method

First author, year	Country	Disease	Detection method	No.of subjects (CXCR4+/CXCR4-)	Median age(range)	Median follow-up(months)	Period	Cutoff value detection
Wang,2011	China	Non-small cell lung cancer	IHC	208(117/91)	60(35-76)	67	2002-2004	Staining intensity score and percentage of positive cells, the threshold score was 2
Scala,2007	Italy	Uveal melanoma	IHC	25(7/18)	62(25-84)	68	1984-2003	Percentage of positive tumor cells. The threshold score for CXCR4 was 50%
Oda,2007	Japan	Ovarian cancer	IHC	52(20/32)	58(36-77)	26.1	1998-2004	Staining intensity score,cutoff value was 2
He,2013	China	Gastric cancer	IHC	97(43/54)	ND	ND	2000-2005	Staining intensity score
Zobair,2013	China	Non-small cell lung cancer	IHC	125(62/63)	59(37-80)	ND	2003-2011	Percentage of positive tumor cells. The threshold score for CXCR4 was 10%
Popple,2012	UK	Ovarian cancer	IHC	241(214/27)	61(24-90)	167	1984-1997	Tumors were classified by H scores and assessed for high, moderate, low and negative CXCR4 expression
Pils,2007	Austria	Ovarian cancer	IHC	119(64/55)	58.6(27.6-87.2)	43.7	ND	Staining intensity score,cutoff value was 2
Jiang,2006	China	Epithelial ovarian cancer	IHC	44(26/18)	51(43-60)	37	1999-2003	Staining intensity score and percentage of positive cells, the threshold score was 1
Kaifi,2005	Germany	Esophageal Cancer	IHC	136(75/61)	59.5	28	1992-2003	Percentage of positive tumor cells. The threshold score for CXCR4 was 20%
Sasaki,2009	Japan	Esophageal Cancer	IHC	207(174/33)	64(36-92)	42	1987-1998	Staining intensity score,cutoff value was 1
Lu,2011	China	Esophageal Cancer	IHC	127(92/35)	59(39-77)	54	2005-2009	Staining intensity score and percentage of positive cells, the threshold score was 2
Lu,2014	China	Esophageal Cancer	IHC	154(101/53)	62(35-85)	ND	2006-2010	Staining intensity score and percentage of positive cells, the threshold score was 2
Andre,2009	France	Breast cancer	IHC	794(92/702)	ND	120	1989-1995	Staining intensity score and percentage of positive cells, the threshold score was 1
Liu,2010	China	Breast cancer	IHC	200(110/90)	51(37-74)	88	1997-2004	Staining intensity score and percentage of positive cells, the threshold score was 6
Chu,2010	US	Triple negative breast cancer	WB	151(30/121)	50	37	1988-2006	CXCR4 expression was defined as high (>6 fold) or low (<6 fold)
Holm,2009	US	Locally advanced breast cancer	WB	54(19/35)	50	30	ND	CXCR4 expression was defined as high (>6.6 fold) or low (<6.6 fold)
Blot,2008	France	Node-negative breast cancer	IHC	194(155/39)	55(29-84)	ND	1991-2001	Percentage of positive tumor cells. The threshold score for CXCR4 was 10%
Salvucci,2006	Germany	Breast cancer	IHC	1382(969/413)	62(26-101)	68	1985-2001	Staining intensity score and percentage of positive cells
Hiller,2011	US	Locally advanced breast cancer	WB	77(22/55)	51	42	1996-2009	CXCR4 expression was defined as high (>6.6 fold) or low (<6.6 fold)
Chu,2011	US	Hormone receptor-positive,node-negative breast cancer	WB	101(22/79)	54	59	1998-2007	CXCR4 expression was defined as high (>6.6 fold) or low (<6.6 fold)
Holm,2007	US	HER 2 negative breast cancer	WB	103(41/62)	53	26	ND	CXCR4 expression was defined as high (>6.6 fold) or low (<6.6 fold)
Hassan,2009	Canada	Breast cancer	IHC	236(139/97)	50	39.6	2000-2003	Staining intensity score and percentage of positive cells,the patients were divided into low, medium, and high expression categories using outcome-derived cut points from X-tile.
Mizell,2009	US	HER-2 negative breast cancer	WB	115(13/102)	51	53	1998-2006	CXCR4 expression was defined as high (>6.6 fold) or low (<6.6 fold)
Kwak,2005	Korea	Gastric cancer	IHC	307(112/195)	ND	ND	1995-2003	Staining intensity score,cutoff value was 2
Reckamp,2009	US	Advanced non-small cell lung cancer	FACS	16(5/11)	73(42-83)	15	2004-2006	2500 cells/ml
Spoo,2007	US	Acute myelogenous leukemia	FACS	90(58/32)	62.5(18-93)	13	2001-2004	cutoff value for mean fluorescence intensity ratios was 5
Li,2014	China	Epithelial ovarian cancer	IHC	124(75/49)	ND	ND	2004-2007	Staining intensity score and percentage of positive cells, the threshold score was 5

First author, year	Country	Disease	Detection method	No.of subjects (CXCR4+/CXCR4-)	Median age(range)	Median follow-up(months)	Period	Cutoff value detection
An,2014	China	Clear cell renal cell carcinoma	IHC	225(110/115)	ND	62	1999-2006	Staining intensity score and percentage of positive cells, the threshold score was 2
Li,2013	France	Clear cell renal cell carcinoma	IHC	104(68/36)	64.5(34-86)	79.5	1999-2005	Percentage of positive tumor cells. The threshold score for CXCR4 was 25%
Li,2011	China	Advanced renal cell carcinoma	IHC	117(59/58)	59(16-85)	51	2001-2005	Staining intensity score,cutoff value was 2
Zhang,2013,II	China	Myelodysplastic syndrome	FACS	81(40/41)	52(12-77)	ND	2006-2011	cutoff value for mean fluorescence intensity ratios was 29.34
D'Alterio,2010	Italy	Renal cancer	PCR	170(107/63)	68(35-82)	ND	1999-2007	Percentage of positive tumor cells. The threshold score for CXCR4 was 20%
Ahn,2013	US	Acute myeloid leukemia	IHC	53(26/27)	60(32-83)	14.5	ND	Percentage of positive tumor cells. The threshold score for CXCR4 was 10%
D'Alterio,2014	Italy	Rectal cancer	IHC	68(33/35)	ND	64	ND	Percentage of positive tumor cells. The threshold score for CXCR4 was 50%
Shiozaki,2013	Japan	Vulvar cancer	IHC	30(19/11)	66(23-86)	ND	1999-2010	Percentage of positive tumor cells. The threshold score for CXCR4 was 50%
Rombouts,2004	netherlands	Adult acute myeloid leukemia	FACS	90(55/35)	44(16-88)	ND	ND	Percentage of positive labeled cells. The threshold score for CXCR4 was 36.4%
Xiang,2009	China	Hepatocellular carcinoma	IHC	181(91/90)	51(12-86)	20	1999-2007	Staining intensity score,cutoff value was 2
Kim,2005	US	Colorectal Cancer	PCR	35(18/17)	76(41-97)	ND	ND	Patients were dichotomized as having high or low *CXCR4* expression based on the median of all normalized stage I/II *CXCR4* expression values
Yao,2011	China	Gallbladder cancer	IHC	72(50/22)	60(32-84)	29.6	1995-2005	Staining intensity score and percentage of positive cells, the threshold score was 2
Jung,2011	Korea	Prostate cancer	IHC	57(36/21)	64(51-76)	39	2001-2008	Staining intensity score,cutoff value was 2
Spano,2004	France	Non-small cell lung cancer	IHC	61(17/44)	60.6(38-84)	75.6	1987-1999	Staining intensity score and percentage of positive cells. Positive staining was defined as score of 6 or 9 (any slide with >50% of the cells expressing staining with intermediate or strong intensity).
Marechal,2009	Belgium	Pancreatic adenocarcinoma	IHC	71（39/32）	64.5(39-81)	ND	1998-2006	Staining intensity score and percentage of positive cells, the threshold score was 3
Wagner,2009	US	Lung cancer	IHC	154(47/107)	67	26.4	ND	Staining intensity score,cutoff value was 2
Sekiya,2012	Japan	Clear cell carcinoma of the ovary	IHC	42(21/21)	52(27-69)	ND	1993-2006	Staining intensity score and percentage of positive cells, the threshold score was 3
Bao,2013	China	Multiple myeloma	FACS	227(98/129)	58(32-84)	ND	2006-2012	Percentage of positive labeled cells. The threshold score for CXCR4 was 20%
Liu,2014	China	Colorectal Cancer	IHC	92(56/36)	61(30-86)	65	2005-2007	Staining intensity score,cutoff value was 2
Koishi,2006	Japan	Esophageal cancer	IHC	24(13/11)	60(44-78)	ND	1996-2003	Staining intensity score,cutoff value was 2
Gebauer,2011	Germany	Pancreatic adenocarcinoma	IHC	249(215/34)	63(32-87)	ND	1994-2005	Percentage of positive labeled cells. The threshold score for CXCR4 was 20%
Akashi,2008	Japan	Metastatic prostate cancer	IHC	52(18/34)	73(54-87)	ND	1986-1999	Percentage of positive labeled cells. The threshold score for CXCR4 was 50%
Fanelli,2012	Brazil	Gastric cancer	IHC	104(85/19)	65(20-88)	ND	1998-2006	Staining intensity score and percentage of positive cells, the threshold score was 3
Cabioglu,2007	US	Inflammatory breast cancer	IHC	44(18/26)	49(29-73)	46.5	1994-2002	Percentage of positive labeled cells. The threshold score for CXCR4 was 50%
Chen,2013,I	China	Triple-negative breast cancer	IHC	75(53/22)	50(29-83)	ND	2000-2008	Staining intensity score,cutoff value was 2
Almofti,2004	Japan	Oral squamous cell carcinoma	IHC	59(35/24)	ND	ND	1985-2000	Percentage of positive labeled cells. The threshold score for CXCR4 was 25%

First author, year	Country	Disease	Detection method	No.of subjects (CXCR4+/CXCR4-)	Median age(range)	Median follow-up(months)	Period	Cutoff value detection
Almofti,2004	Japan	Oral squamous cell carcinoma	IHC	59(35/24)	ND	ND	1985-2000	Percentage of positive labeled cells. The threshold score for CXCR4 was 25%
Lee,2009,II	Korea	Gastric cancer	IHC	221(108/113)	ND	ND	2000-2003	Staining intensity score,cutoff value was 2
Zhang,2012	China	Stage II-III colon cancer	IHC	125(74/51)	61.8	78	2001-2005	Staining intensity score and percentage of positive cells, the threshold score was 4
Ying,2012	China	Gastric cancer	IHC	26(13/13)	56(29-85)	70.5	2004	Staining intensity score and percentage of positive cells, the threshold score was 4
Lin,2011	China	Osteosarcoma	IHC	56(39/17)	18(7-67)	33.5	2002-2006	Percentage of positive tumor cells. The threshold score for CXCR4 was 20%
Chen,2013,II	China	Bilateral breast cancer	IHC	33(22/11)	48(21-89)	44.5	2000-2008	Staining intensity score and percentage of positive cells, the threshold score was 1
Longo-Imedio,2005	Spain	Primary melanoma	IHC	40(14/26)	61(21-89)	32	ND	Percentage of positive tumor cells. The threshold score for CXCR4 was 20%
Segawa,2009	Japan	Nasopharyngeal carcinoma	IHC	76(41/35)	56(19-85)	ND	1985-2007	Staining intensity score and percentage of positive cells, the threshold score was 4
Franco,2010	Italy	Melanoma	IHC	32(22/10)	ND	ND	1998-2006	Percentage of positive tumor cells. The threshold score for CXCR4 was 30%
Yu,2013	China	Triple-negative breast cancer	IHC	148(102/46)	ND	ND	1995-2011	Staining intensity score and percentage of positive cells, the threshold score was 4
Saigusa,2010	Japan	Rectal cancer	PCR	53(16/37)	62(37-78)	48	2001-2008	positive:detectable
Kodama,2007	Japan	Cervival cancer	IHC	174(110/64)	46(25-67)	56.5	2001-2006	Percentage of positive labeled cells. The threshold score for CXCR4 was 50%
Wang,2005	China	Nasopharyngeal carcinoma	IHC	194(88/106)	45(25-70)	ND	2000-2004	Staining intensity score,cutoff value was 4

Suppl. fig. 2: Methodological quality of all studies based on the Newcastle-Ottawa scale for assessing the quality of cohort trials

First author, year	Representativeness of exposed cohort	Selection of non-exposed cohort	Asertainment of expoure	Outcome not present at start of study	Comparability based on the design or analysis	Asertainment of outcome	Follow-up long enough for outcomes	Adequacy of follow-up	Total score
Chen,2014	1	1	1	1	1	1	1	1	8
Minamiya,2010	1	1	1	0	2	1	1	0	7
Mirisola,2009	1	1	1	0	0	1	1	0	5
Lee,2009,I	1	1	1	1	1	1	1	1	8
Clatot,2011	1	1	1	1	0	1	1	0	6
Albert,2012	1	1	1	1	0	1	1	1	7
Konoplev,2013	1	1	1	1	2	1	1	1	9
Gockel,2006	1	1	1	0	0	1	1	1	6
Wang,2012	1	1	1	1	2	1	1	0	8
Zhang,2013,I	1	1	1	1	2	1	1	0	8
Speetjens,2009	1	1	1	1	2	1	1	1	9
Konoplev,2007	1	1	1	1	2	1	1	1	9
Parker,2012	1	1	1	1	2	1	1	0	8
Oda,2009	1	1	1	1	2	1	1	0	8
Andre,2006	1	1	1	0	1	1	1	0	6
Yopp,2012	1	1	1	1	2	1	1	1	9
Katayama,2005	1	1	1	1	2	1	1	0	8
Scala,2005	1	1	1	1	2	1	1	0	8
Ottaiano,2006	1	1	1	1	2	1	0	0	7
Otsuka,2011	1	1	1	1	0	1	1	0	6
Wang,2011	1	1	1	1	2	1	1	0	8
Scala,2007	1	1	1	1	0	1	1	0	6
Oda,2007	1	1	1	1	2	1	1	0	8
He,2013	1	1	0	1	2	1	1	1	8
Zobair,2013	1	1	1	1	2	1	1	0	8
Popple,2012	1	1	1	1	1	1	1	1	8
Pils,2007	1	1	1	1	1	0	1	0	6
Jiang,2006	1	1	1	1	0	1	1	0	6
Kaifi,2005	1	1	1	1	1	1	1	0	7
Sasaki,2009	1	1	1	0	0	1	1	0	5
Lu,2011	1	1	1	1	0	1	1	0	6
Lu,2014	1	1	1	1	0	1	1	1	7
Andre,2009	1	1	1	1	0	1	1	0	6
Liu,2010	1	1	1	1	0	1	1	0	6
Chu,2010	1	1	1	0	0	1	1	0	5
Holm,2009	1	1	1	1	0	1	1	0	6
Blot,2008	1	1	1	1	0	1	1	0	6
Salvucci,2006	1	1	0	1	2	1	1	0	7
Hiller,2011	1	1	1	1	0	1	1	0	6

First author, year	Representativeness of exposed cohort	Selection of non-exposed cohort	Asertainment of expoure	Outcome not present at start of study	Comparability based on the design or analysis	Asertainment of outcome	Follow-up long enough for outcomes	Adequacy of follow-up	Total score
Chu,2011	1	1	1	1	0	1	0	0	5
Holm,2007	1	1	1	1	0	1	0	0	5
Hassan,2009	1	1	1	1	0	1	0	1	6
Mizell,2009	1	1	1	1	0	1	1	0	6
Kwak,2005	1	1	1	0	0	1	0	1	5
Reckamp,2009	1	1	1	1	0	1	1	0	6
Spoo,2007	1	1	1	0	2	1	1	1	8
Li,2014	1	1	1	1	0	1	1	1	7
An,2014	1	1	1	1	1	1	1	1	8
Li,2013	1	1	1	0	2	1	1	1	8
Li,2011	1	1	1	0	0	1	1	0	5
Zhang,2013,II	1	1	1	1	2	1	1	0	8
D'Alterio,2010	1	1	1	1	2	1	1	0	8
Ahn,2013	1	1	1	0	2	1	1	1	8
D'Alterio,2014	1	1	1	1	2	1	1	0	8
Shiozaki,2013	1	1	1	1	2	1	1	0	8
Rombouts,2004	1	1	1	1	0	1	1	1	7
Xiang,2009	1	1	1	1	0	1	1	1	7
Kim,2005	1	1	1	1	0	1	1	1	7
Yao,2011	1	1	1	1	0	1	1	0	6
Jung,2011	1	1	1	1	0	1	1	1	7
Spano,2004	1	1	1	0	0	1	1	0	5
Marechal,2009	1	1	1	1	0	1	1	1	7
Wagner,2009	1	1	1	1	0	1	1	1	7
Sekiya,2012	1	1	1	0	0	1	1	1	6
Bao,2013	1	1	1	1	0	1	1	1	7
Liu,2014	1	1	1	1	0	1	1	1	7
Koishi,2006	1	1	1	1	0	1	1	0	6
Gebauer,2011	1	1	1	1	0	1	1	1	7
Akashi,2008	1	1	1	1	0	1	1	0	6
Fanelli,2012	1	1	1	1	0	1	1	1	7
Cabioglu,2007	1	1	1	1	0	1	1	1	7
Chen,2013,I	1	1	1	1	0	1	1	0	6
Almofti,2004	1	1	1	1	0	1	1	0	6
Lee,2009,II	1	1	1	1	0	1	1	0	6
Zhang,2012	1	1	1	0	0	1	1	0	5
Ying,2012	1	1	1	0	0	1	1	1	6
Lin,2011	1	1	1	1	0	1	1	0	6
Chen,2013,II	1	1	1	1	0	1	1	0	6

First author, year	Representativeness of exposed cohort	Selection of non-exposed cohort	Asertainment of expoure	Outcome not present at start of study	Comparability based on the design or analysis	Asertainment of outcome	Follow-up long enough for outcomes	Adequacy of follow-up	Total score
Chen,2013,II	1	1	1	1	0	1	1	0	6
Longo-Imedio,2005	1	1	1	1	0	1	1	0	6
Segawa,2009	1	1	1	1	0	1	1	1	7
Franco,2010	1	1	1	1	0	1	1	1	7
Yu,2013	1	1	1	1	0	1	1	1	7
Saigusa,2010	1	1	0	1	0	1	1	0	5
Kodama,2007	1	1	1	1	0	1	0	1	6
Wang,2005	1	1	1	1	0	1	1	0	6

Suppl. table 3 List of adjustment factors employed in all studies included in current meta-analysis.

AJCC, American joint committee on cancer; BMI, body mass index; BRE grade, Elston and Ellis grade; CCR7, C-C chemokine receptor type 7; CEBPA, CCAAT/enhancer-binding protein alpha; CRM, circumferential resection margin; CXCL 12, C-X-C motif chemokine 12; CXCR 7, C-X-C chemokine receptor type 7; ECOG, eastern cooperative oncology group; ER, estrogen receptor; FIGO, the international federation of gynecological oncologists; FLT 3, fms-like tyrosine kinase 3; HER2, human epidermal growth factor receptor 2; ITD, internal tandem duplication; LDH, lactate dehydrogenase; LRP, leucine responsive protein; MMP-9, matrix metallopeptidase 9; NPM1, nucleophosmin 1; PR, progesterone receptor; SLN, sentinel lymph nodes; STAT 3, signal transducer and activator of transcription 3; TRG, tumor regression grade; VEGF, vascular endothelial growth factor; WBC, white blood cells; WHO, world health organization; YB 1, Y box binding protein 1; ND, no data.

First author, year	country	Adjustment for Covariates
Chen,2014	Germany	TNM stage,pathological grade,epithelial-mesenchymal transition related genes
Minamiya,2010	Japan	Age,sex,differentiation grade,tumor size,lumph node metastasis
Mirisola,2009	Germany	ND
Lee,2009,I	Korea	Tumor size,lymph node metastasis,TNM stage,MMP-9
Clatot,2011	France	ND
Albert,2012	France	ND
Konoplev,2013	US	Age,history of antecedent hematologic disorder,thrombocytopenia,serum creatnine level,serum albumin level,NPM1
Gockel,2006	Germany	ND
Wang,2012	China	Age,sex,AJCC stage,lymph node status,metastasis,histologic variant, Fuhrman's grade
Zhang,2013,I	China	Age,gender,WHO grade,TNM stage
Speetjens,2009	Netherlands	Age,sex,tumor location,TNM stage,microsatellite status
Konoplev,2007	US	Age,sex,race,performance status,incorporation of cytarabine in the therapeutic regimen,antecedent hematological
Parker,2012	US	Age,ER,PR,HER 2 status,TNM stage,WHO grade
Oda,2009	Japan	Age,sex,site,size,depth,mitosis,necrosis,histologic grade,AJCC stage,MIB-LI,VEGF
Andre,2006	France	Treatment
Yopp,2012	US	Age,sex,clinic risk score,margin,distribution,CCR7,CXCL12 expression
Katayama,2005	Japan	Age,sex,primary site,tumor differentiation,clinical stage
Scala,2005	Italy	Age,sex,Breslow, SLN status,presence of ulceration
Ottaiano,2006	Italy	Age,sex,AJCC stage,VEGF expression
Otsuka,2011	Canada	ND
Wang,2011	China	Age,tumor size,smoking,lymph node status,stage,tumor classificaiton,pathological stage,STAT3,VEGF status
Scala,2007	Italy	ND
Oda,2007	Japan	Age,stage,grade,tumor size,YB-1,P-gp,p-Akt,LRP expression
He,2013	China	Age,sex,location,differentiation,Lauren classification,tumor size,TNM stage,distant metastasis

First author, year	country	Adjustment for Covariates
Zobair,2013	China	Age,clinic stage and treatment
Popple,2012	UK	FIGO stage,macroscopic residual disease,adjuvant therapy
Pils,2007	Austria	Histology,FIGO stage,grade,HER2,SDF-1
Jiang,2006	China	ND
Kaifi,2005	Germany	Lymph node metastasis,histologic grade,lymph node micrometastasis,bone marrow micrometastasis
Sasaki,2009	Japan	ND
Lu,2011	China	ND
Lu,2014	China	ND
Andre,2009	France	ND
Liu,2010	China	ND
Chu,2010	US	ND
Holm,2009	US	ND
Blot,2008	France	ND
Salvucci,2006	Germany	Age, T stage,BRE grade,number of positive lymph nodes, ER and PR status
Hiller,2011	US	ND
Chu,2011	US	ND
Holm,2007	US	ND
Hassan,2009	Canada	ND
Mizell,2009	US	ND
Kwak,2005	Korea	ND
Reckamp,2009	US	ND
Spoo,2007	US	Age,sex,WBC,LDH,cytogenetic abnormalities
Li,2014	China	ND
An,2014	China	Tumor size,T stage,N stage,M stage,TNM stage,Fuhrman grade,tumor necrosis,ECOG status

First author, year	country	Adjustment for Covariates
Li,2013	France	Age,sex,T stage,N stage,M stage,Fuhrman,ECOG status,necorsis
Li,2011	China	ND
Zhang,2013,II	China	Age,sex,hemoglobin,WBC,platelet,karyotype and marrow blast
D'Alterio,2010	Italy	Age,sex,clinic presentation,AJCC stage,Fuhrman grade,Lymphonodes,CXCR7
Ahn,2013	US	Age,sex,WHO classificaiton,NPM1,CEBPA,FLT3-ITD,FLT3-D835
D'Alterio,2014	Italy	Age,sex,histology,TRG Mandard,T stage,CRM,N status,CXCL12
Shiozaki,2013	Japan	Age,FIGO stage,histology
Rombouts,2004	netherlands	ND
Xiang,2009	China	ND
Kim,2005	US	ND
Yao,2011	China	ND
Jung,2011	Korea	ND
Spano,2004	France	ND
Marechal,2009	Belgium	ND
Wagner,2009	US	ND
Sekiya,2012	Japan	ND
Bao,2013	China	ND
Liu,2014	China	ND
Koishi,2006	Japan	ND
Gebauer,2011	Germany	ND
Akashi,2008	Japan	ND
Fanelli,2012	Brazil	ND
Cabioglu,2007	US	ND
Chen,2013,I	China	ND

First author, year	country	Adjustment for Covariates
Chen,2013,I	China	ND
Almofti,2004	Japan	ND
Lee,2009,II	Korea	ND
Zhang,2012	China	ND
Ying,2012	China	ND
Lin,2011	China	ND
Chen,2013,II	China	ND
Longo-Imedio,2005	Spain	ND
Segawa,2009	Japan	ND
Franco,2010	Italy	ND
Yu,2013	China	ND
Saigusa,2010	Japan	ND
Kodama,2007	Japan	ND
Wang,2005	China	ND
